# The impact of online store specifications on enhancing the attractiveness of customer perception of the product: An analytical study of the opinions of a sample of Iraqi virtual store customers

**DOI:** 10.12688/f1000research.175115.1

**Published:** 2026-04-30

**Authors:** Sadia Awid Awni, Ahmed Abbas Hammadi, Imad Ali Mahmood Al-halboosi, Hisham Jadallah Mansour Shakaterh, Doaa Salman, Ayat Muhammad Nabil Wahib Ababneh, Andriy Stavytskyy, Farouq Ahmad Faleh Alazzam, Rafat Hisham Shakaterh

**Affiliations:** 1 Technical Institute of Management, Middle Technical University, Baghdad, Baghdad Governorate, Iraq; 2 Business Administration Department, University of Fallujah, Al-Fallujah, Al Anbar Governorate, Iraq; 3 Business Administration Department, Iraqi University, Baghdad, Iraq; 4 Middle East University Faculty of Law, Amman, Amman Governorate, Jordan; 5 University for Modern Sciences & Arts(MSA), Egypt, Egypt; 6 Faculty of Financial and Administrative Sciences, Amman, Jordan; 7 University of Kyiv, Ukraine, Ukraine; 8 Department of private Law, United Arab Emirates University, Al Ain, Abu Dhabi, United Arab Emirates; 9 Jadara University Faculty of Law, Irbid, Irbid Governorate, Jordan

**Keywords:** online store specifications, customer perception appeal, online stores.

## Abstract

The research aims to explain the role of the specifications of some Iraqi electronic stores in enhancing the attractiveness of the customer’s perception of the product, as the recent literature that dealt with the research variables recorded good results that enhance the goal of the research, but there is still a lot to investigate and learn about, especially in an environment that differs from the environments of previous literature, and I follow The research used a descriptive analytical approach. The research community was represented by all customers dealing with electronic stores in Baghdad Governorate. The research sample included (350) customers for the period from 1-3-2023 to 1-7-2023. In order to analyze the data, several statistical methods were used: During the (Smart Pls.4) program, the research reached several results, the most important of which is the existence of a correlation and influence of the specifications of the online store in enhancing the attractiveness of the customer’s perception of the product.

## 1. Introduction

Online shopping has become very popular because of the comfort and ease it provides to the buyer, in addition to competitive offers in price and offers on various product information to create and enhance customer satisfaction Since the primary goal of any organization is to create and maintain a large customer base, companies in the age of digital must upgrade their websites, maintain the degree of quality that the customer expects, and continue to develop them in conjunction with smart applications of information and communications technology and the Internet ^7^ The organization’s presentation of its products through its website has become a prevailing trend left by the Corona crisis that has swept the world and has enabled it to diversify and develop its offers in a way that meets the renewed needs and desires of customers. This requires it to closely follow digital developments in this field, in addition to continuous monitoring of customer satisfaction, which contributes to the formation of a more efficient and effective electronic marketing strategy, in light of the existence of evidence indicating that many consumers who search websites, They have the intention to make purchases and the purchase may be abandoned if consumers find that these sites are contrary to their desires. The quality of the online store is related to a certain part or level of consumer satisfaction, as it determines the extent of success or failure of the website. In the end, the deciding point is the extent to which the intention to buy from the same store increases in the future and repeatedly, with recommendations for other customers to buy through comments in the store All of this prompted researchers to delve into this research and apply it in an environment where electronic marketing is considered almost new. ^15^

## 2. Research problem

The traditional view prevailing in our local environment is that every purchase online is not trustworthy because the customer does not touch or see the product in its real reality at first, which raises negative doubts about the product in the customer’s purchasing intention, as recent statistics indicate that approximately 30% Online users do not commit to shopping online due to low trust. In light of most companies’ efforts to gain customers, satisfy them, and repeat purchases of their products, these companies have resorted to thinking of new ways to market their products electronically through their websites to attract and satisfy customers by adhering to the quality of their website specifications. They are currently the most widely used, and sales competition through them has become great between companies, and with it, sales growth has increased rapidly and significantly In order for Iraqi online shopping stores to become like any other country in which electronic marketing has flourished, whether through social networking sites or through active electronic pages that have contributed somewhat to the spread of the culture of selling, this research was conducted on a number of local online shopping stores to find out the extent of awareness of the owners of these sites of the specifications of their marketing stores from the point of view of the customers dealing with them and in a way that contributes to enhancing the customer’s attractiveness towards purchasing Hence, the research problem will be raised through the following questions:

1. Do customers’ perceptions of search variables vary by gender and age?
2. How aware are customers of the specifications of the shopping stores searched?
3. What is the role of the specifications of the shopping stores studied in enhancing the attractiveness of the customer’s perception of the product?

## 3. Research objectives

1. Learn about the most important specifications that make the online store more desirable for purchase from the point of view of the customers being searched.
2. Find out which customers buy the most and by gender
3. Knowing the type of relationship between the specifications of the online store and the attractiveness of the customer’s perception of the product.
4. Knowing the extent to which the specifications of the online store affect the attractiveness of the customer’s perception of the product.

## 4. Importance of research

1- Applying advanced technological innovation in electronic buying and selling operations in the local environment, which enhances confidence in dealing with shopping stores electronically.
2- Taking advantage of speed and time in shopping operations, which helps in providing products in large quantities and at reasonable prices compared to regular shopping.

## 5. Research plan and hypothesis

**Figure 1.**
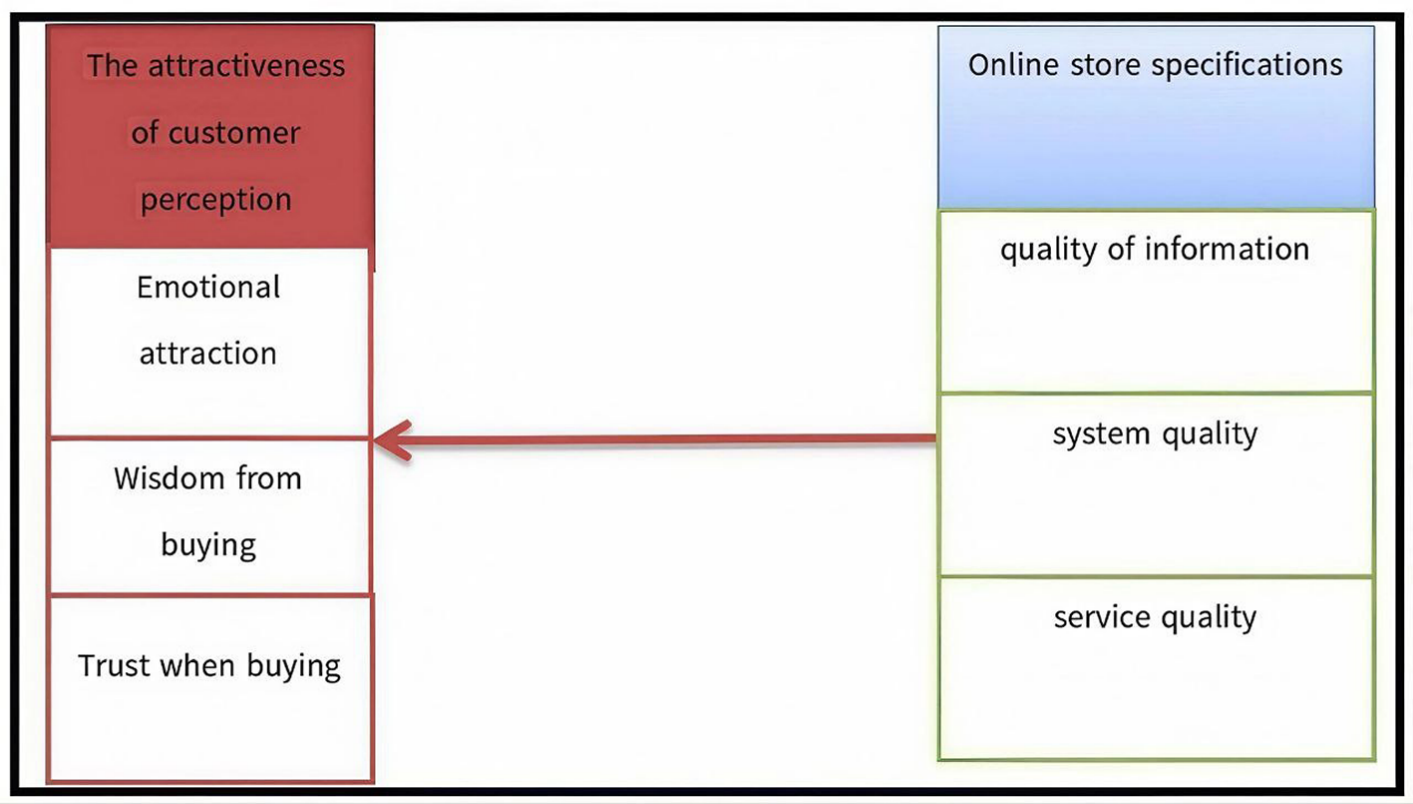
Hypothetical diagram of the research.

Figure 1 below illustrates the conceptual framework of the study, showing the nature of the relationships between the main and sub-research variables:

**HO1:** The number of purchases from the searched online stores for the searched customers varies according to the gender variable.

**HO2:** There is a statistically significant effect (α ≤ 0.05) of the specifications of the online store in terms of its individual dimensions (quality of information, quality of the system, quality of services (in enhancing the attractiveness of the customer’s perception in terms of its dimensions) emotional attraction, wisdom in purchasing, confidence when purchasing (in Iraqi online stores. The following branches out from this hypothesis **:**

**HO2–**1: There is a statistically significant effect (α ≤ 0.05) of information quality in enhancing the attractiveness of customer perception in the Iraqi online stores studied.

**HO2–2:** There is a statistically significant effect (α ≤ 0.05) of system quality in enhancing the attractiveness of customer perception in the Iraqi online stores studied.

**HO2–3:** There is a statistically significant effect (α ≤ 0.05) of the quality of services in enhancing the attractiveness of customer perception in the Iraqi online stores studied.

**HO4:** There is a statistically significant effect (α ≤ 0.05) of the specifications of the online store in terms of its combined dimensions (quality of information, quality of the system, quality of services (in enhancing the attractiveness of the customer’s perception in terms of its dimensions) emotional attraction, wisdom in purchasing, confidence when purchasing)in Iraqi online stores.

## 6. Theoretical framework and previous studies

### 1- Specifications of the online store

Customers are often looking for products that make their lives easier and less expensive, and with the development of marketing and promotion methods that have accompanied technical developments, marketing and promotion via websites has become the most widespread and popular among customers. ^20^ With the ease of marketing through online stores and choosing various products with discounts and high quality, shopping through online stores has become a feature of the current era and the most attractive and opens great horizons for customers to choose what they. ^11^ An online store is a system with globally accepted standards for storing, retrieving and displaying information in the digital environment, and the word specification for a store website is a necessary concept in the world of online shopping ^14, 18^ believes that businesses cannot attract a relatively wide range of visitors without a high-quality online store, as the quality of the online store’s specifications is characterized by its ability to allow customers to achieve their goals and their willingness to return to visit the store to perform the purchasing experience on an ongoing basis. An online store is defined as a technical term for a variety of files with different formats such as text, images, audio, and video. ^12^

There are concerns about the needs behind the research interest in measuring the quality of online store specifications. In many previous research attempts, the concept of online store quality was initially limited to ease of use and usability, which are a feature of store design and are defined here as the extent to which a product can be used by specific users to achieve goals efficiently and effectively. ^13^ Since the online store interface is an essential user interface for online marketing, it is important to evaluate the quality of the store’s specifications and who the customers will visit the store. ^1^ believes that stores must now study efficiency and satisfaction as essential factors for the success of the online store and identify factors that attract customers in terms of not delaying the download of offers, ease of navigation in the online store, interaction and response, and quality of content. ^14^ pointed out that the specifications of the online store that are decisive for the success of e-commerce are represented by measuring customer satisfaction, through his purchasing behavior from the store, as there are two types of intentions related to the success of the online store, the first is the initial intention to purchase and the second is the intention to purchase continuously, and they are closely related to converting and retaining customers. Better-designed online stores can increase actual online purchases and the intention to repurchase again in the future. The success of online stores occurs when the specifications of the online store are developed to provide the highest level of quality compared to other competing stores, and the result of this is when customers choose this store as the most preferred store, and this leads to the possibility of improving business performance in the store chosen or preferred by customers. ^20^ believe that other basic goals contribute to satisfaction, including the convenience of online shopping, the online environment, the relationship between employees and customers, and the value of the product. ^19, 15, 1^ believe that the process of customer satisfaction digitally requires quality in the system and quality of the information and services provided, which will be addressed as specifications for the online store:

**First: Quality of information:-**

The quality of information is one of the most important specifications of an online store, as information about products must be sufficient and always updated on store websites ^20^ The content of the information also has a direct impact on the customer’s opinion and evaluation of the effectiveness of the online store. ^4^ Information quality is defined as the degree to which a customer believes that information on a store’s website possesses the attributes of content, accuracy, form ^6^,and timeliness. ^16^ Empirical results support the observation that information quality positively affects user satisfaction ^14^ and perceived benefit. High quality information is positively associated with the success of a store’s website, as customers are fully aware of the quality of the products and services offered on the store’s website. As such, customers seek information that allows them to distinguish between a seller of high-quality goods and services and a seller of low-quality goods or services. Because there may be many online stores that provide information about similar products and services, what may attract customers to a particular online store to make purchases are the features of the information provided by that online store. ^18^

**Second: Perceived quality of service:-**

Perceived service quality is defined as the degree to which a customer believes that an online store is responsive and interactive. ^3^ clear about security and privacy policies, and effective in search and comparison capabilities, ^8^ Customer service on the web can take many forms, such as responding to inquiries and providing search and comparison capabilities. Tools that improve customer service include dedicated web pages and a list of frequently asked questions These tools can be classified into interaction and response service categories, as interaction includes the ability to customize the appearance, look, and content of the site in addition to providing interaction with the customer, while response is concerned with providing feedback to customers ^1, 9^ believe that online stores must prove that the information they provide benefits customers and will not be used in any way that harms customers’ privacy concerns. Ensuring that the online store’s website is secure for transactions is essential to allay fears that others will not intercept the information customers send. Quality of service is considered the comprehensive support provided by the online store owner on the basis of guarantee, empathy and response. At the same time, it is a vital dimension of the website that has a direct impact on purchasing intentions and customer satisfaction by responding to customer requests and simplifying two-way communication. This explains electronic social communication, which may lead to customer satisfaction and loyalty.

**Third: System quality:**

System quality is the quality of information system processing, which evaluates ease of use, functionality, availability, flexibility, reliability and response time and is considered the key aspect in achieving effective and secure electronic marketing. ^1^ System quality greatly affects the success of an online store, as factors such as website responsiveness, system usefulness, suitability, reliability, and availability are important aspects that must be taken into consideration during the system design phase in order to provide optimal system quality to the customer, as considering these aspects enhances customers’ purchasing intentions from the online store. ^10^ According to, ^4^ an online store is a system with globally accepted standards for storing, retrieving and displaying information electronically. An online store website provides two types of functions: first, information sources that can provide different types of information that customers need. This information can be catalogs, databases, archives, bulletins, etc., and second, an interactive service that can serve activities between customers and servers.

### 2. The attractiveness of the customer’s perception of the product

One of the most important principles of customer attractiveness and perception of the product is choosing the best among the available alternatives, and thus dissonance decreases and good purchasing behavior arises after realizing the value of the product they will buy with the extent of their inclination towards the brand. Therefore, ^4^ identified the dimensions of customer perception attractiveness, which are (emotional attraction, wisdom in purchasing, confidence when purchasing) based on the study: ^19^

1.
**Emotional attraction:** The attraction between internal feelings and expected or actual emotional expressions through customer interactions leads to the experience of emotional attraction, so it was found that emotional attraction is positively related to various customer outcomes such as repeat purchase behavior, sharing of the product, feeling of customer achievement, and well-being. ^1^^–^^15^ believes that since the compatibility between purchases and customer emotion is a positive feeling, most customers take action during the purchase process, and more specifically, marketers must attach emotional content to brands, as the more positive experiences and (emotional) moments that the marketer shares with the brand, the more likely customers are to become loyal to the brand.
2.
**Confidence when purchasing:** Sometimes a customer has a constant or temporary anxiety about the products purchased through online stores. Here, “purchase anxiety” can be defined by ^18^ as “the customer’s recognition after purchasing may have been influenced by his own beliefs by sales staff. This final dimension recognizes the potential cognitive discrepancy resulting from changing the customer’s attitude through the influence of the sales representative. In other words, was the customer’s decision affected by advertising and promotion by online stores. ^17^ stated that whenever a customer makes a decision, they will have some degree of anxiety about the purchase, and this will create cognitive dissonance. This means that they will have doubts and anxiety about the choice they made, because the lost alternatives had certain desirable attributes and the selected choice had some undesirable elements that the customer must now accept When there is dissonance, the customer will try to downplay or avoid the negative aspects of his decision and reinforce the positive elements. ^5^ pointed out that the sales representative works to clarify the characteristics that characterize new products, so they found that new product buyers read advertisements that support their choices and stay away from reading those that conflict with their decisions significantly more than non-buyers, and summarized Chapanis&Chapanis said that customers who had recently purchased products through online stores experienced cognitive dissonance and tried to reduce the resulting anxiety by selectively exposing themselves to supportive virtual advertising.
3. Previous studies
  The study ^2^ aimed to test the factors affecting online shopping in light of the Covid-19 pandemic in the Kingdom of Saudi Arabia. The five main factors identified from the literature review related to online shopping were analyzed and studied: product variety, convenience, payment method, trust, and psychological factors in the Saudi context. The research collected data online through a previously tested tool Which was directed to Saudi consumers online through various electronic tools such as email and social media platforms, the results of the statistical analysis showed that only three factors have a significant direct impact on online shopping in light of the Covid-19 pandemic. These factors are product diversity, payment method, and psychological factors While convenience and trust have failed to significantly impact consumers’ online shopping decisions during the COVID-19 pandemic, both factors have been less important to consumers. Online shopping has become more popular among people during the COVID-19 pandemic, and the result will help e-commerce companies better meet consumer demands by adjusting their strategies. The **study** ^19^ aimed to present a validated model that provides an explanation for the phenomenon of online shopping and that integrates and expands on previous work by incorporating new premises. This study used a multi-method approach to develop a comprehensive model of consumers’ online shopping behaviors. To achieve this, in addition to a literature review, qualitative data were collected to identify a wide range of possible antecedent factors, and then using a longitudinal questionnaire, The Consumer Shopping Intentions and Behaviors Model was validated on 9,992 consumers, and the results showed that the main motivating factors for online shopping are compatibility, impulsive purchasing behavior, value awareness, risk, local shopping, shopping pleasure, and browsing pleasure. As for the **study**, ^18^ which showed that the emergence of e-marketing has revolutionized consumer behavior, profoundly affecting how individuals interact with brands, make purchasing decisions, and engage in commerce, this research paper summarizes the available literature to study the multifaceted effects of e-marketing on consumer purchasing behavior, and through a comprehensive analysis, key insights are extracted about the different ways in which e-marketing strategies, such as personal recommendations, affect Targeted advertising and interactive experiences on consumer attitudes, preferences, and decision-making processes in the digital world. Furthermore, the paper explores the role of social media platforms, technological developments, and changing consumer expectations in shaping the e-marketing landscape. It also discusses ethical considerations, challenges, and future directions for research and practice to provide a comprehensive understanding of the dynamic relationship between e-marketing and consumer behavior. Overall, this review contributes to a deeper understanding of the complexities involved in dealing with the e-marketing environment It stresses the importance of aligning marketing strategies with consumers’ preferences and evolving trends in the digital age. The **study** ^4^ aimed to analyze the impact of website quality and brand image on the consumer’s purchasing decision, considering trust as an intermediate variable (case study on Bukalapak.com). The aim of the study was to survey the opinions of Bukalapak.com customers, who numbered 100 respondents. The sample was selected using the purposeful random sampling method, while the analytical method used is structural equation modeling (SEM). The results showed: (1) Website quality has a positive and significant impact on the purchasing decision, (2) Brand image has a positive and significant impact on the purchasing decision, (3) Trust has a positive and significant impact on the purchasing decision, (4) Website quality has a positive and significant impact on trust, and (5) Brand image has a positive and significant impact on trust.
  The research gap in enhancing the attractiveness of customer perception of a product is one of the important issues that needs in-depth study. Despite the rapid growth of e-commerce, there is still a lack of comprehensive understanding of how online store design, content, and ease of use affect customers’ perception of the attractiveness of products. There is a need to explore the factors that make the online shopping experience more attractive, and their impact on purchasing decisions There is also a need to identify knowledge gaps related to the psychological aspect of customers and how they interact with the various elements of the store, which contributes to improving marketing strategies and developing electronic platforms.

## 7. Research methodology

### 1. Study community and sample

The study population consisted of customers of ten selected online stores in Baghdad Governorate – Iraq, as shown in the Table 1 below:

**Table 1.** Electronic stores.

T	Online store name	Employment	Link/means of communication	Brand
1	Miswag store	Miswag is the first online shopping site in Iraq and the largest retail site by value in the Iraqi market. The Maswag website was established in 2014 and was designed and completed by Iraqi skills at the hands of website programming specialists and commercial experts.	https://miswag.com/more/about-us	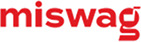
2	i-Digi store	It is an Iraqi online store that provides a distinctive shopping experience for customers in Iraq by providing a large collection of high-quality mobile accessories at competitive prices while ensuring the comfort of customers and focusing on providing their needs through easy access to goods and delivering them as quickly as possible. Idigi Store is an Iraqi online store that is part of the e-marketing company iDigi Marketing (registered in Canada – Ontario.	https://i-digistore.com/about-us/	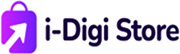
3	KoLSHZIEN	.kolsh Zien store is considered one of the largest and most reliable Iraqi stores that sells products of all categories. Kolsh Zien provides everything you need from electronics, computer and mobile accessories, and women’s supplies such as perfumes, makeup, and others. Kalash Zain provides savings and delivery services to anywhere in Iraq, inside and outside Baghdad, in the rest of the Iraqi governorates	https://share.google/YtL7WnWCQd77jReEn	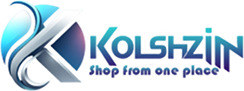
4	Orisdi	Orisdi is considered one of the leading platforms in the field of online shopping in Iraq, as it is distinguished by providing a comprehensive and integrated shopping experience. The site offers a wide range of products including fashion, electronics, home appliances and cosmetics, with a particular focus on high-quality local and international products.	https://orisdi.com/	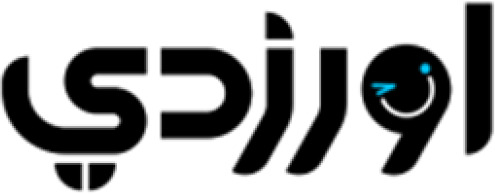
5	**Elryan**	Elryan is a specialized online shopping destination in Iraq, with a special focus on electronics, health and beauty supplies, fashion, and modern accessories. The site offers a carefully selected collection of clothing, shoes and bags, targeting youth and modern fashion. What distinguishes Elryan is its regular updates that keep pace with the latest international fashion trends	www.najma-store.com	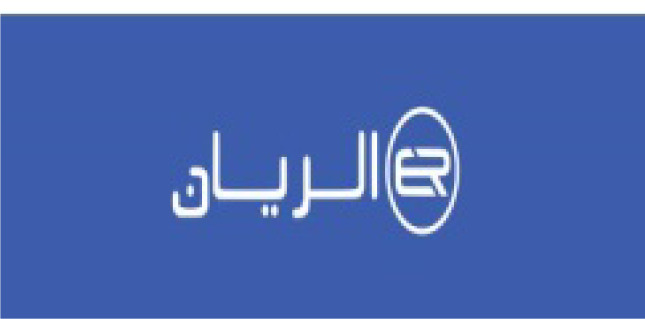
6	**Naram**	Naram stands out as an online shopping site in Iraq, specializing in providing healthcare and beauty products. The site features a wide range of medical products, skin care products, and nutritional supplements from trusted international brands. What distinguishes Naram is its focus on providing educational information about products	https://naram.com/	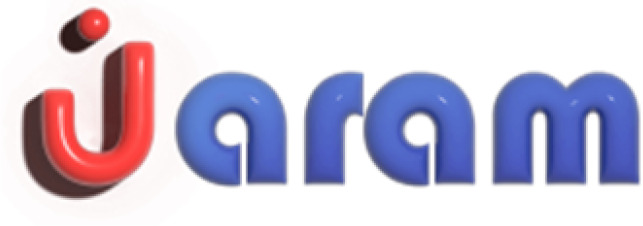
7	**Mishmish**	The Mishmish application stands out as an innovative online shopping platform in Iraq, specializing in providing a quick and easy shopping experience via smartphones. The application features a simple and attractive user interface, which facilitates browsing and purchasing. Mishmish focuses on offering a selection of essential everyday products, including groceries, personal care products, and small electronics.	https://mishmish.app/	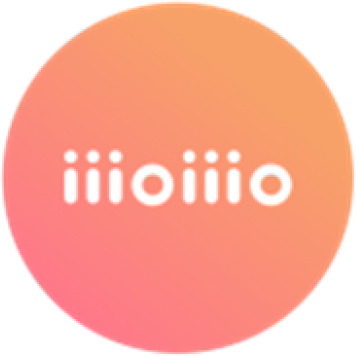
8	**Jum3a**	Jum3a website is distinguished by being a unique platform for online shopping in Iraq, as it focuses on providing the best weekly offers and discounts. The site takes its name from jum3a, as it offers special and special offers on this day. Jumaa covers a variety of products, with a particular focus on electronics and home appliances. What distinguishes the site is the smart alert system that informs users of the latest offers and discounts. In addition, Jumaa offers a loyalty program that gives regular shoppers additional benefits. With easy-to-use interface and reliable delivery service,	https://jum3a.com/	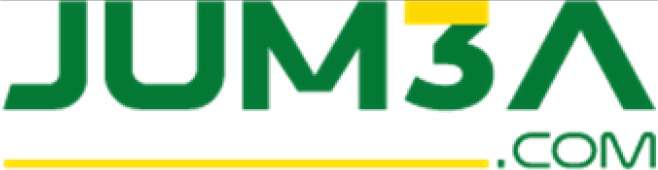
9	Ubuy	It is one of the best online shopping stores in the country. We offer a wide variety of international industrial products	https://share.google/o6HF1jvUPJWn2ufsI	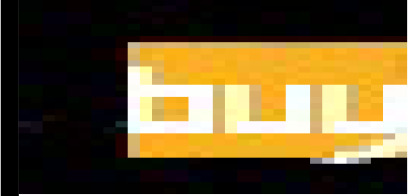
10	**Bazzaar –baghdad**	Your favorite online shopping store. We offer you the best products at reasonable prices and high quality, such as shoes and others	https://www.bazaar-baghdad.com/	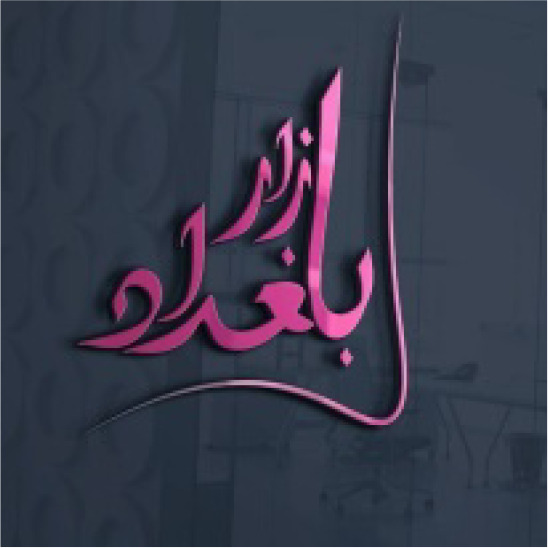

The number (750) represents the total volume of electronically active customers during the time period from February 3, 2025 to February 20, 2025. As for the study sample, it was selected using the simple random sampling method By distributing a questionnaire online through various electronic tools such as email and social media platforms, this method provides efficiency in reaching participants and ease in collecting and analyzing data (Creswell, 2014).

For the purpose of determining the appropriate sample size, the Yamane equation (1967) was relied upon to determine the sample size in a simple random sample, which takes into account the size of the statistical population and the acceptable error rate. Based on this equation, the sample size for a population of (750) individuals, with a margin of error of (5%), is calculated as follows:

n=N/(1+N(e^2))=750/(1+750(0.05^2))≈261

Accordingly, the minimum sample size required is at least 261 participants. Since the number of actual responses obtained during the data collection period amounted to (350) completed questionnaires, this size exceeds the minimum number required, which enhances the reliability and accuracy of the results and gives the study greater statistical power.

## 8. Practical application

### First: Evaluating the quality of the standards

**1 – Variable model of online store specifications**

**Figure 2.**
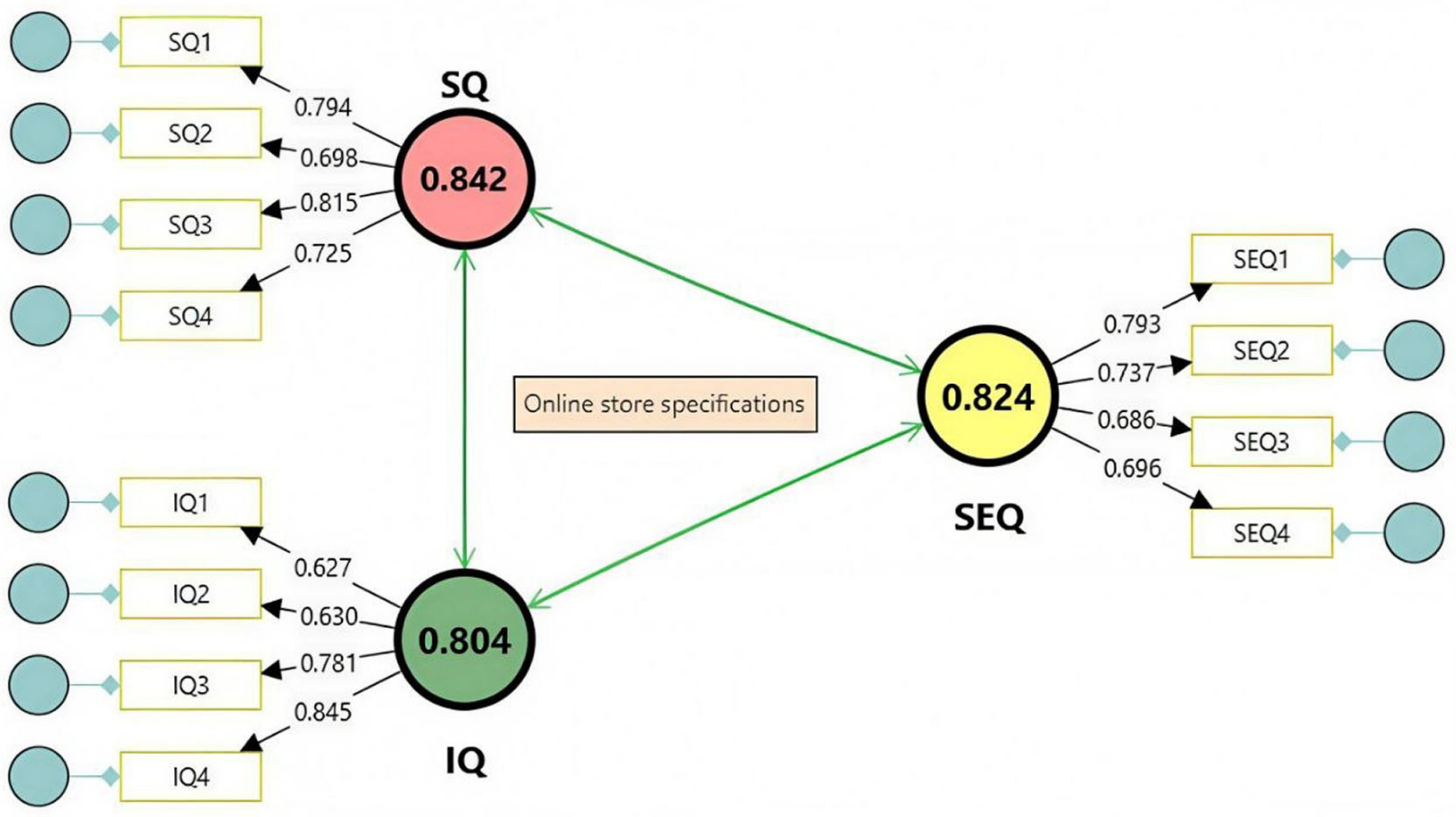
Electronic store specifications model.

Figure 2, which represents the questions and dimensions of the online store specifications model, shows that it consists of three basic dimensions, with (4) questions for each dimension. Table 2 shows the (CR) values for the online store specifications variable, all of which are within acceptable limits, as they ranged between (0.845–0.812), which is a good indicator and indicates the stability of the scale The results showed a high stability of the dimensions of the variable scale of the electronic store specifications, as shown by the value of the Alfacronbach coefficient, which ranged between (0.842–0.804), as it is clear that it is greater than (0.70), and this indicates the presence of high stability The results showed that the values of the average extracted variance (AVE) for the online store specification variable are all acceptable, as they ranged between (0.577–0.528), which is greater than the value (0.50), as it indicates that the sub-dimensions contribute significantly to explaining the total variance of the online store specification variable, and therefore the model is considered more reliable in explaining the relationships between the dimensions of the variable.

**Table 2.** Estimates of the dimensions of the online store specifications variable.

(AVE)	Composite reliability	Cronbach’s alpha)	P values	T values	Estimate	Dimensions	questions
0.577	0.845	0.842			0.794	**quality of information**	SQ1
			0.000	11.889	0.698		SQ2
			0.000	14.225	0.815		SQ3
			0.000	12.417	0.725		SQ4
0.528	0.812	0.804			0.627	**quality of system**	IQ1
			0.000	8.979	0.63		IQ2
			0.000	10.588	0.781		IQ3
			0.000	11.179	0.845		IQ4
0.532	0.820	0.824			0.793	**quality of services**	SEQ1
			0.000	12.915	0.737		SEQ2
			0.000	11.860	0.686		SEQ3
			0.000	12.069	0.696		SEQ4

Source: Smart Pls.4

Table 2 shows the values of the estimates, which ranged between (0.845–0.627), as it is clear that all the questions are influential. It is also clear from the values of (t), which ranged between (14.225–8.979), which is also greater than the tabular value of (t), which is (1.984), which is a sufficient indicator for adopting the model in the final form in subsequent analyses.

**2- The attractiveness of the customer’s perception of the product**

**Figure 3.**
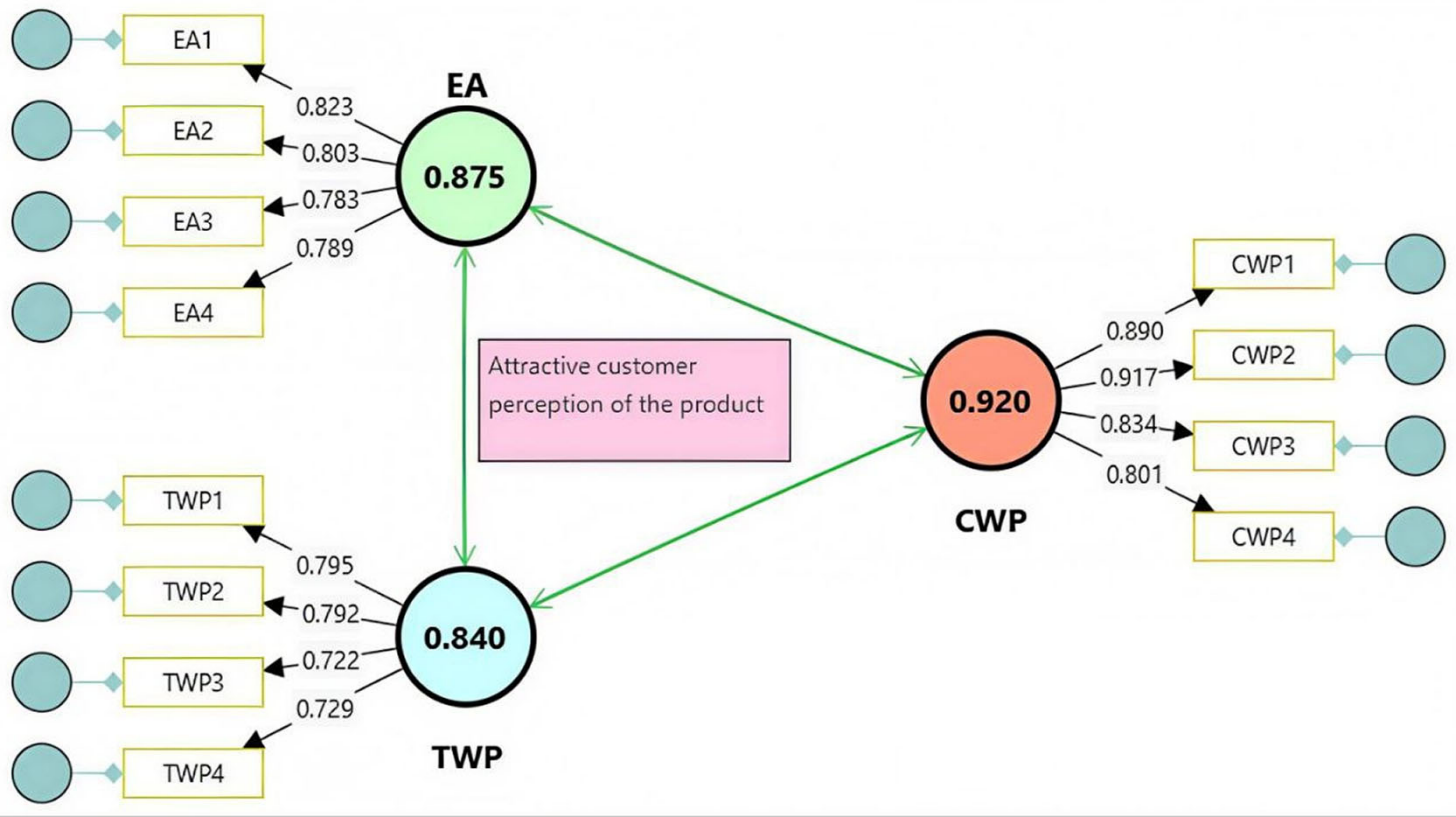
Model of the attractiveness of the customer’s perception of the product.

Figure 3, which represents the questions and dimensions of the customer perception attractiveness model for the product, shows that it consists of three basic dimensions with (4) questions for each dimension. Table 2 shows the values of (CR) for the customer perception attractiveness variable for the product, all of which are within acceptable limits as they ranged between (0.916–0.845), which is a good indicator and indicates the stability of the scale The results showed a high stability of the dimensions of the variable scale, the attractiveness of the customer’s perception of the product, as shown by the value of the Alfacronbach coefficient, which ranged between (0.92–0.84), as it is clear that it is greater than (0.70), and this indicates that there is also a high stability The results showed that the values of the average extracted variance (AVE) for the customer perception attractiveness variable for the product were all acceptable, as they ranged between (0.743–0.578), which is greater than the value (0.50), as it indicates that the sub-dimensions contribute significantly to explaining the total variance of the customer perception attractiveness variable for the product, and therefore the model is considered more reliable in explaining the relationships between the two dimensions of the variable.

Table 3 shows the values of the estimates, which ranged between (0.917–0.722), as it is clear that all the questions are influential. It is also clear from the values of (t), which ranged between (23.049–12.841), which is also greater than the tabular value of (t), which is (1.984), which is a sufficient indicator for adopting the model in the final form in subsequent analyses.

**Table 3.** Estimates of the dimensions of the customer perception attractiveness variable for the product.

(AVE)	Composite reliability	Cronbach’s alpha)	P values	T values	Estimate	Dimensions	questions
0.640	0.875	0.875			0.823	**emotional attraction**	EA1
			0.000	15.404	0.803		EA2
			0.000	14.869	0.783		EA3
			0.000	15.029	0.789		EA4
0.578	0.845	0.840			0.795	**wisdom in purchasing**	TWP1
			0.000	14.431	0.792		TWP2
			0.000	12.841	0.722		TWP3
			0.000	13.002	0.729		TWP4
0.743	0.916	0.920			0.89	**confidence when purchasing**	CWP1
			0.000	23.049	0.917		CWP2
			0.000	18.933	0.834		CWP3
			0.000	17.551	0.801		CWP4

Source: Smart Pls.4

### Second: Descriptive analysis of research variables

**1- Specifications of the online store**

It is noted from Table 4 that the highest general arithmetic mean was reached at the dimension of (quality of information), as it reached (3.613) and at a good level, as its standard deviation reached (0.772) and a coefficient of variation (21.37), as this dimension came at level (1) in terms of relative importance The lowest general arithmetic mean was at the (quality of service) dimension, which reached (3.565), with a good level, a standard deviation of (0.820), and a coefficient of variation of (23.00), as this dimension came at level (3) in terms of relative importance The average was also general arithmetic at the dimension (system quality), reaching (3.597), with a good level, a standard deviation of (0.805), and a coefficient of variation of (22.39), as this dimension came at level (3) in terms of relative importance Overall, the online store specifications variable achieved a mathematical average of (3.592) at a good level and with a standard deviation of (0.725), as the coefficient of variation reached (20.18), as it came in (first) order in terms of relative importance.

**Table 4.** Descriptive statistics for research variables and dimensions.

relative importance	CV	S	M	Dimensions of search variables
**2**	22.39	0.805	3.597	**quality of system**
**1**	21.37	0.772	3.613	**quality of information**
**3**	23.00	0.820	3.565	**quality of services**
**First**	20.18	0.725	3.592	**Online store specifications**
**1**	23.05	0.853	3.701	**emotional attraction**
**2**	24.02	0.877	3.652	**wisdom in purchasing**
**3**	26.46	0.938	3.547	**confidence when purchasing**
**Second**	22.67	0.824	3.633	**The attractiveness of the customer’s perception of the product**

Source:SPSS V.28

2- The attractiveness of the customer’s perception of the product

It is clear from Table 4 that the highest general arithmetic mean was reached at the dimension of (emotional attraction), as it reached (3.701) and at a good level, as its standard deviation reached (0.853) and a coefficient of variation (23.05), as this dimension came at level (1) in terms of relative importance The lowest general arithmetic mean was at the dimension of (confidence when buying), which reached (3.547), with a good level, a standard deviation of (0.938), and a coefficient of variation of (26.46), as this dimension came at level (3) in terms of relative importance The average was also general arithmetic at the dimension (wisdom of purchase), which amounted to (3.652), with an average level, a standard deviation of (0.877), and a coefficient of variation of (24.02), as this dimension came at level (2) in terms of relative importanceOverall, the variable of attractiveness of the customer’s perception of the product achieved a mathematical average of (3.633) at a good level and with a standard deviation of (0.824), as the coefficient of variation reached (22.67), which came in the (second) sequence in terms of relative importance.

### Third: Testing research hypotheses

**−1 Test the first hypothesis**

**H01: The number of purchases made from the online stores searched for the searched customers varies by gender.**

In this paragraph, the Mann-Whitney test will be conducted to test the variation in the number of purchases from the searched online stores for the searched customers by gender, As shown in Table 5 and as follows:

**Table 5.** Testing the variation in purchases from online stores by gender.

Asymp. Sig.	Mann-Whitney	Mean Rank	Division or category	feature
**0.442**	**6430.1**	**134.09**	**Male**	**gender**
		**140.83**	**Female**	

The value of (Asymp. Sig.) Which amounted to (0.442), which is greater than (0.05), which indicates that there is no significant difference between the times of purchases from electronic stores with regard to gender.

**Figure 4.**
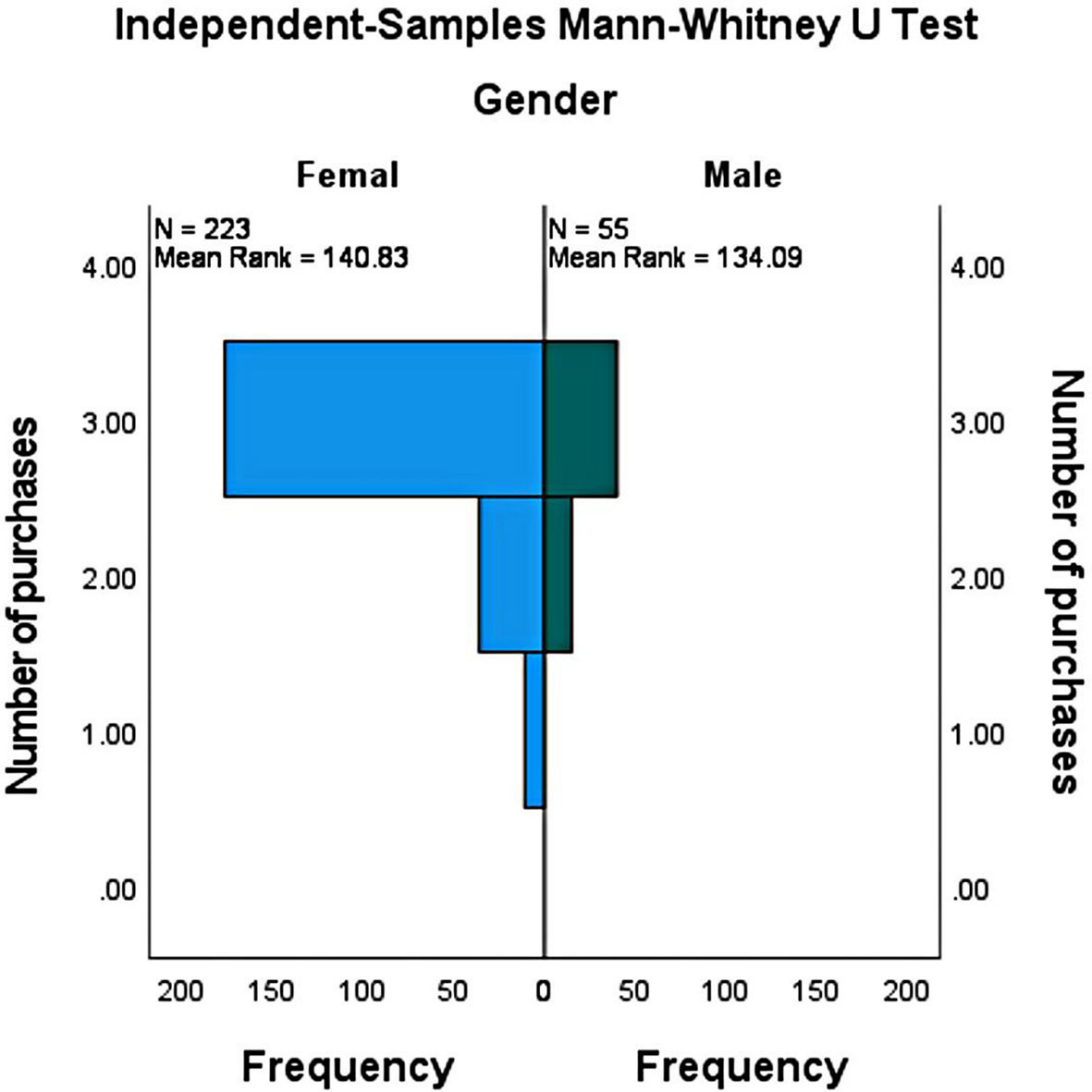
Frequencies of number of purchases by gender.

**Figure 5.**
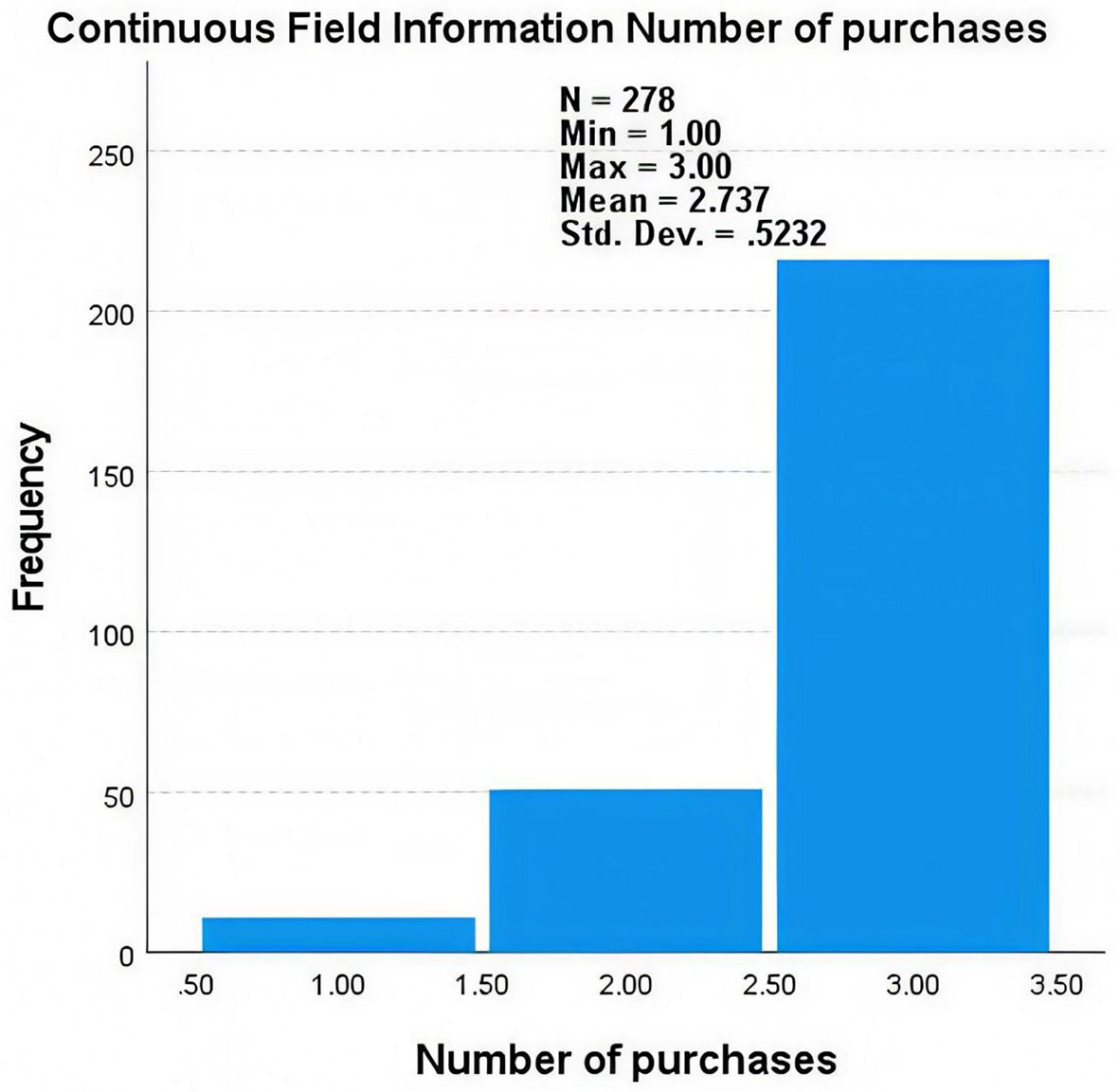
Frequencies of number of purchases.

Figure 4 shows the frequencies of the number of purchases by gender using the Mann-Whitney test, showing slight differences in the average ranks between males and females:

Figure 5 shows, as shown above, the frequencies of the number of purchases, and shows that most participants made actual purchases, with an overall average of approximately 2.74.

**2- Testing the second hypothesis**

**H02: There is a significant effect of the specifications of the online store on the attractiveness of the customer’s perception of the product in the Iraqi online stores studied.**

**Figure 6.**
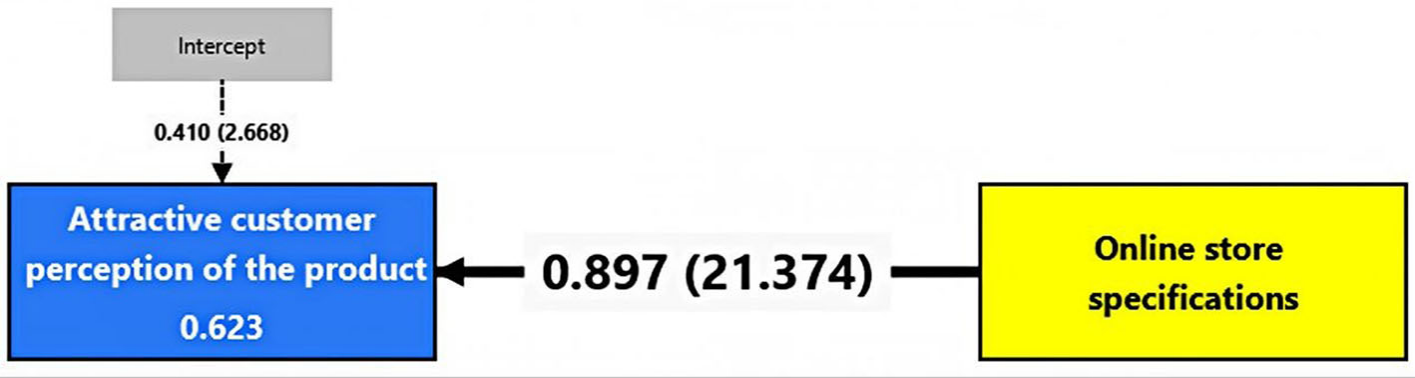
Analysis of the impact between the specifications of the online store on the attractiveness of the customer’s perception of the product.

It is clear from Table 6 and Figure 6 that the value of (F) extracted among the specifications of the online store in the attractiveness of the customer’s perception of the product was recorded at (456.858), which is (greater) than the tabular (F) of (3.94) at the significance level of (0.05), and this result provides sufficient support to accept the alternative hypothesis, which states that. (There is a morally significant effect of the specifications of the online store on the attractiveness of the customer’s perception of the product) and this indicates the existence of a morally significant effect of the specifications of the online store on the attractiveness of the customer’s perception of the product, as the specifications of the online store were able to explain (62%) of the variables that occur in the attractiveness of the customer’s perception of the product. The extracted value of (t) for the online store specifications variable was also recorded as (21.374). It is greater than the tabular value (t) of (1.984) at the significance value of (0.05). This indicates that the significance of (β) for the variable of the electronic store specifications is proven, as it is clear from the value of (β) that increasing the specifications of the electronic store by one unit will lead to an increase in the attractiveness of the customer’s perception of the product by (89%).

**Table 6.** Analysis of the impact between the specifications of the online store on the attractiveness of the customer’s perception of the product.

dependent variable	Sig	(t)	(F)	(R ^2^)Adj	(R ^2^)	R	independent variable		
**The attractiveness of the customer’s perception of the product**	**0.000**	**21.374**	**456.858**	0.622	0.623	0.790	0.410	**(α)**	**Online store specifications**
							0.897	**(β)**	

**3- Testing the third hypothesis**

**HO3: There is a statistically significant effect (α ≤ 0.05) of the specifications of the online store in terms of its dimensions (quality of information, quality of the system, quality of services)in enhancing the attractiveness of the customer’s perception in terms of its dimensions(emotional attraction, wisdom in purchasing, confidence when purchasing)in Iraqi online stores, as it is clear from Table 7 the following:**

1- The value (F) extracted between the dimensions of the online store specifications in terms of the attractiveness of the customer’s perception of the product achieved (156.942, 386.616, 438.421) respectively, which is greater than the tabular value (F) of (3.94) at the significance level of (0.05), and based on that, the decision is as shown in Table 6
2- The value of the correlation coefficient (R) recorded a value of (0.602, 0.764, 0.783), and this indicates that there is a difference in the correlation value between the dimensions of the online store specifications and the variable of the attractiveness of the customer’s perception of the product, as it turns out that the highest correlation value was at the dimension (quality of service), as it reached (0.783) The results also showed that the lowest correlation value was at the (system quality) dimension, reaching (0.602)
1- It achieved the value of Adj(R2) (0.360, 0.582, 0.612) and this indicates that there is a difference in the interpretation of the dimensions of the electronic store specifications for the variable of the attractiveness of the customer’s perception of the product. It turns out that the highest explanatory percentage was at the dimension of (quality of information), as Ma explained (58%) of the variables that occur in the variable of the attractiveness of the customer’s perception of the product The lowest explanatory percentage was at the (system quality) dimension, as Ma explained (36%) of the variables that occur in the variable of attractiveness of the customer’s perception of the product.
2- The extracted value of (t) for the marginal slope coefficient between the dimensions of the electronic store specifications in the attractiveness of the customer’s perception of the product was (12,528, 19,663, 20,938), respectively. It is greater than the tabular value (t) of (1.984) at the significance level of (0.05), and this indicates the proof of the significance of the marginal slope coefficient for the dimensions of the electronic store specifications.
3- It is clear from the value of (β) for all dimensions, which is (0.616, 0.815, 0.787) respectively, indicating that there is a variation in the impact force of the dimensions of the online store specifications on the variable of the attractiveness of the customer’s perception of the product, as it is clear that the highest impact force was at the dimension (quality of information), as increasing this dimension by one unit will lead to an increase in the variable of the attractiveness of the customer’s perception of the product by (81%) It is also clear that the least impact force was at the dimension (system quality), as increasing this dimension by one unit will lead to an increase in the variable of attractiveness of the customer’s perception of the product by (61%).
Table 8 Analysis of the impact of the dimensions of the online store specifications on the attractiveness of the customer’s perception of the product.

**Table 7.** Sub-hypotheses of the effect between the dimensions of online store specifications on the attractiveness of the customer’s perception of the product.

Resolution	Hypothesis	Hypothesis symbol
**Proof of the alternative hypothesis**	**There is a significant effect of the system quality dimension on the attractiveness of customer perception of the product**	H21
**Proof of the alternative hypothesis**	**There is a significant effect of the information quality dimension on the attractiveness of customer perception of the product**	H22
**Proof of the alternative hypothesis**	**There is a significant effect of the quality of service dimension on the attractiveness of customer perception of the product**	H23
**0**	**Number of acceptable null (zero) hypotheses**	
**3**	**Number of acceptable alternative hypotheses**	

**Table 8.** Analysis of the impact between the dimensions of the online store specifications on the attractiveness of the customer’s perception of the product.

Sig	T	F	Adj (R ^2^)	R ^2^	R	B	Α	Dimensions of online store specifications	
0.000	12.528	156.942	0.360	0.363	0.602	0.616	1.418	**quality of system**	The attractiveness of the customer’s perception of the product
0.000	19.663	386.616	0.582	0.583	0.764	0.815	0.689	**quality of information**	
0.000	20.938	438.421	0.612	0.614	0.783	0.787	0.827	**quality of services**	

Source:SPSS V.28

**3- Testing the fourth hypothesis**

**HO4: There is a statistically significant effect (α ≤ 0.05) of the specifications of the online store in terms of its combined dimensions (quality of information, quality of the system, quality of services) in enhancing the attractiveness of the customer’s perception in terms of its dimensions (emotional attraction, wisdom in purchasing, confidence when purchasing) in Iraqi online stores.**

**Figure 7.**
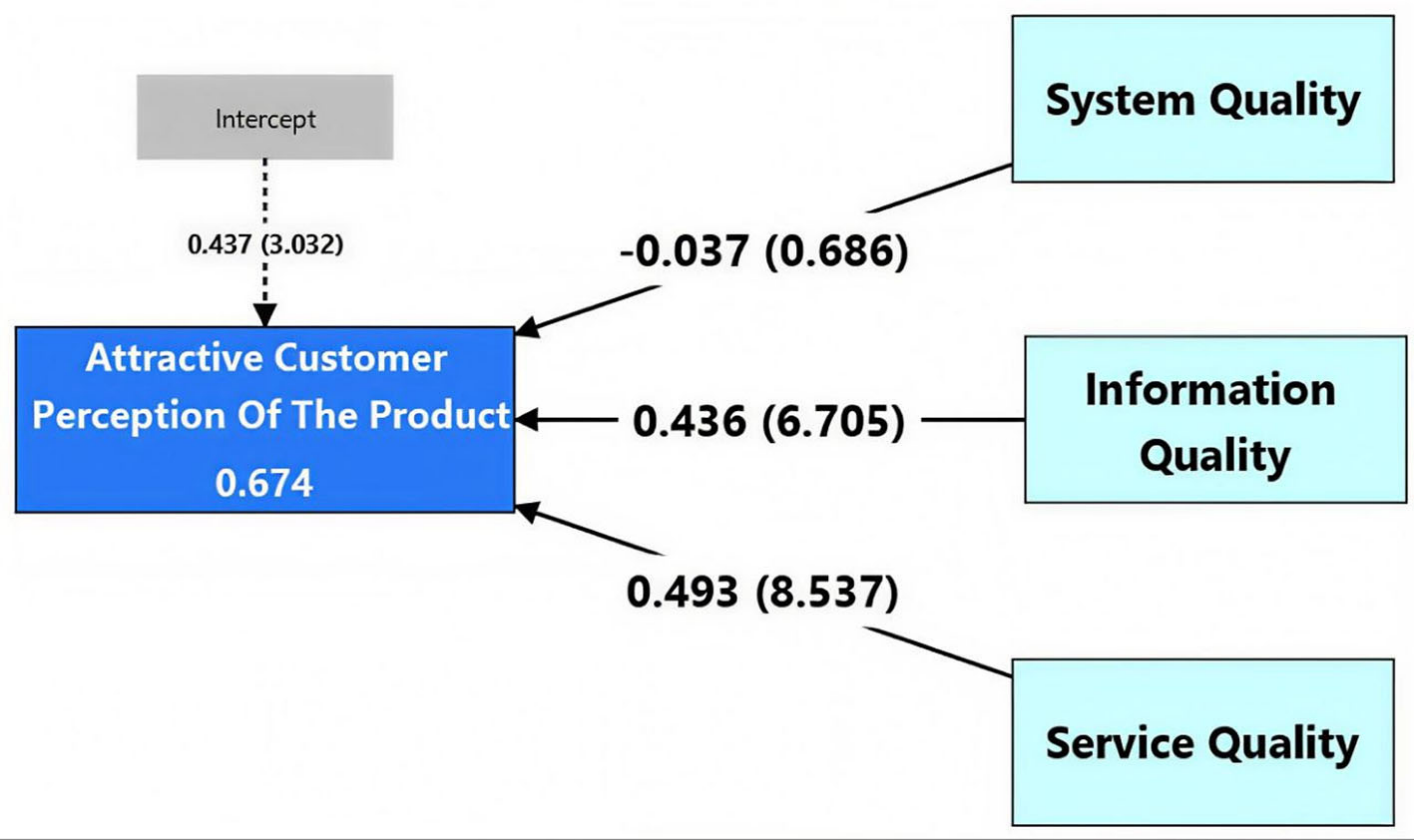
Impact analysis of the dimensions of online store specifications, combining the attractiveness of the customer’s perception of the product.

Table 9 and Figure 7 indicate the results of the impact analysis of the dimensions of the online store specifications combined in the attractiveness of the customer’s perception of the product, as the extracted value (F) achieved a value of (188,878) and indicates the presence of a significant impact between the online store specifications together in the attractiveness of the customer’s perception of the product It is clear from the value of R2)Adj) that the dimensions of the online store specifications together were able to explain (67%) of the changes that occur in the attractiveness of the customer’s perception of the product. It also appears from the extracted value (t) of (6,705, 8,537) respectively that it is greater than the tabular value (t) of (1,984) and indicates that the effect of the parameter (β) for the two dimensions (quality of information, quality of service) is a real effect, as increasing the effect by one unit will lead to an increase in the attractiveness of the customer’s perception of the **product by (43%, 49%) Respectively, as for the effect after (system quality), the results showed that it has no significant effect on the attractiveness of the customer’s perception of the product.**

**Table 9.** Analysis of the impact of the dimensions of the online store specifications together on the attractiveness of the customer’s perception of the product.

Sig.	(F)	(R ^2^)Adj	(R ^2^)	(R) المتعدد	Sig.	(t)	(β)	(α)	Dimensions of online store specifications
**0.000**	188.878	0.670	0.674	0.821	0.493	0.686-	0.037-	**0.437**	**quality of system**
					0.000	6.705	0.436		**quality of information**
					0.000	8.537	0.493		**quality of services**
**2.70**					**(F) Tabular**				
**1.984**					**(t) Tabular**				
**Number of acceptable dimensions (Influencer) = 3**									
**Unacceptable number of dimensions (unaffecting) = 1**									

Source:SPSS V.28

## 9. Discuss the results

The research reached several results, the most important of which is the existence of two relationships and an effect of the specifications of the online store in enhancing the attractiveness of the customer’s perception of the product. The results of the multiple effect showed that the dimensions (quality of information and quality of service), together, were able to explain (67%) of the changes that occur in the attractiveness of the customer’s perception of the product. As for the effect of the dimension (system quality), the results showed that it has no significant effect on the attractiveness of the customer’s perception of the product, and this may indicate that system quality, despite its technical importance as a basic factor in ensuring the efficient operation of platforms or systems, It did not directly contribute to enhancing the customer’s attractive impression of the product because the customer often views system quality as a basic requirement that is supposed to be present in any system or service, and not an additional feature that can increase the attractiveness of the product. Therefore, the presence of a system that works efficiently and easily is a necessary but insufficient condition to form the attractiveness of product perception, as other variables such as information quality, service quality, added value play a role Or innovation plays a more influential role in creating a positive impression on customers.

The results of the current study are consistent with what was stated by ^2^ that some expected dimensions such as comfort and trust were not decisive factors in consumers’ decision, and are also consistent with what was highlighted by ^19, 18^ of a greater role for behavioral and psychological factors and innovative marketing strategies. In contrast, the results of the study differ from what was found by ^4^ which emphasized the importance of website quality (close to the concept of system quality) in enhancing purchasing decisions and trust. This discrepancy can be explained by the fact that the attractiveness of perception is conceptually different from the purchasing decision, in addition to the difference in context (Iraq vs. other markets) and methodology (measuring attractiveness vs. actual purchasing behavior).

Accordingly, the results of the current study highlight that system quality is an expected prerequisite for ensuring continuity of use, but it alone is not sufficient to attract customers. The most influential factors in shaping attractive perception are the quality of information and service, as well as elements of added value and innovation.

## 10. Recommendations

Based on the research results, the current study recommends focusing on the importance of improving the online shopping experience for customers in Iraqi online stores, and developing the design of user interfaces so that they are attractive and easy to use, making it easier for customers to move between departments and find products quickly. It is also essential to improve the quality of the images displayed while providing accurate and detailed product descriptions, as this enhances the customer’s awareness of the product’s attractiveness and increases the likelihood of purchasing. Trust among customers should also be enhanced by providing reviews and opinions from previous users, which helps build the credibility of the online store. It is also recommended to provide multiple and secure payment options, in addition to providing flexible and fast shipping options, which contributes to improving the overall shopping experience. Finally, online stores should focus on providing excellent customer service, including quick and effective support in the event of inquiries or problems. Therefore, the study recommends that the stores surveyed should focus on enhancing the dimensions (quality of service and quality of information) if they want to raise the level of attractiveness of their products, while system quality is viewed as a supporting factor that enhances usage and satisfaction but is not a direct determinant of product attractiveness.

## Ethical considerations

This study involved human participants and was conducted in accordance with accepted ethical research standards and the principles outlined in the Declaration of Helsinki. Ethical approval was obtained from the Scientific Research Ethics Committee, University of Fallujah, Iraq (Approval No. HOF.HUM.2025.001). Written informed consent was obtained from all participants prior to their participation. All participants were informed about the purpose of the study, the voluntary nature of their participation, their right to withdraw at any time without consequences, and the confidentiality of their data.

## Data Availability

The data supporting the findings of this study are openly available in Zenodo at: https://doi.org/10.5281/zenodo.18305448, Hammadi, A. A., Shakhatreh, H. J. M., Awni, S. A., Salman Abdou, D., ababneh, A., Stavytskyy, A., & azzam,. farouq. (2026). The impact of online store specifications on enhancing the attractiveness of customer perception of the product: An analytical study of the opinions of a sample of Iraqi virtual store customers [Data set]. Zenodo. These data are available under the terms of the Creative Commons Zero “No rights reserved” data waiver (CC0 1.0 Public Domain Dedication). This study is an observational survey-based research and follows the STROBE reporting guidelines. No CONSORT or ARRIVE checklists are required, as the study does not involve clinical trials or animal experiments.
