# The impact of online store specifications on enhancing the attractiveness of customer perception of the product: An analytical study of the opinions of a sample of Iraqi virtual store customers

**DOI:** 10.12688/f1000research.175115.2

**Published:** 2026-06-20

**Authors:** Sadia Awid Awni, Ahmed Abbas Hammadi, Imad Ali Mahmood Al-halboosi, Hisham Jadallah Mansour Shakaterh, Doaa Salman, Ayat Muhammad Nabil Wahib Ababneh, Andriy Stavytskyy, Farouq Ahmad Faleh Alazzam, Rafat Hisham Shakaterh

**Affiliations:** 1 Technical Institute of Management, Middle Technical University, Baghdad, Baghdad Governorate, Iraq; 2 Business Administration Department, University of Fallujah, Al-Fallujah, Al Anbar Governorate, Iraq; 3 Business Administration Department, Iraqi University, Baghdad, Iraq; 4 Middle East University Faculty of Law, Amman, Amman Governorate, Jordan; 5 University for Modern Sciences & Arts(MSA), Egypt, Egypt; 6 Faculty of Financial and Administrative Sciences, Amman, Jordan; 7 University of Kyiv, Ukraine, Ukraine; 8 Department of private Law, United Arab Emirates University, Al Ain, Abu Dhabi, United Arab Emirates; 9 Jadara University Faculty of Law, Irbid, Irbid Governorate, Jordan

**Keywords:** online store specifications, customer perception appeal, online stores.

## Abstract

**Background:**

Despite rapid e-commerce growth in emerging markets, approximately 30% of online users in Iraq avoid online shopping due to low trust. Prior research has conflated distinct dimensions of store quality, and no study has specifically investigated how information quality, system quality, and service quality differentially influence customer perceptual attractiveness—a distinct construct comprising emotional attraction, wisdom in purchasing, and confidence when purchasing.

**Objectives:**

This study aims to (1) determine the bivariate and multivariate effects of information quality, system quality, and service quality on customer perceptual attractiveness; (2) test whether purchase frequency varies by gender; (3) assess customer awareness of online store specifications; and (4) identify which specifications contribute most significantly to enhancing product attractiveness.

**Methods:**

A cross-sectional survey was conducted with 350 customers of ten Iraqi online stores in Baghdad Governorate (February 3–20, 2025). Convenience sampling with stratified targeting was employed. Data were analyzed using a two-stage approach: PLS-SEM (SmartPLS 4.0) for measurement model validation (reliability, convergent validity, discriminant validity via HTMT), followed by multiple regression (SPSS V.28) for structural path testing with Variance Inflation Factor (VIF) assessment for multicollinearity.

**Results:**

The measurement model demonstrated acceptable reliability (Cronbach’s α: 0.804–0.920; CR: 0.812–0.916) and convergent validity (AVE: 0.528–0.743). Discriminant validity was established (all HTMT values <0.85). Bivariate analyses showed significant positive effects for all three dimensions (IQ: β = 0.815, p < 0.001; SQ: β = 0.616, p < 0.001; SEQ: β = 0.787, p < 0.001). However, in multivariate analysis, information quality (β = 0.436, p < 0.001, VIF = 2.14) and service quality (β = 0.493, p < 0.001, VIF = 2.08) remained significant, while system quality became non-significant (β = −0.037, p = 0.493, VIF = 1.96). The combined model explained 67% of variance (R ^2^ = 0.674, F = 188.878, p < 0.001). No significant gender difference was found in purchase frequency (Mann-Whitney U = 6430.1, p = 0.442). Customer awareness of store specifications was moderate (M = 3.592, SD = 0.725 on a 5-point scale).

**Conclusions:**

Information quality and service quality function as “motivator factors” that directly enhance customer perceptual attractiveness, while system quality operates as a “hygiene factor”—necessary but not sufficient for differentiation. The suppression of system quality’s effect in multivariate analysis is attributable to multicollinearity among the highly correlated dimensions (r = 0.62–0.71), not to theoretical irrelevance. This represents the first empirical demonstration of Herzberg’s Two-Factor Theory in e-commerce perception research with appropriate multicollinearity controls.

**Scientific Contribution:**

(1) Theoretically, introduces Herzberg’s framework to distinguish hygiene vs. motivator factors in e-commerce; (2) Empirically, provides the first PLS-SEM analysis of e-commerce perception in Iraq with full discriminant validity and multicollinearity reporting; (3) Methodologically, demonstrates the necessity of VIF assessment when interpreting dimension-specific effects in multidimensional quality constructs.

## 1. Introduction

### 1.1 Background

The digital transformation of retail commerce has accelerated dramatically over the past decade, with global e-commerce sales projected to exceed $8 trillion by 2026 ( Statista, 2024). Online shopping offers consumers convenience, price transparency, product variety, and access to global markets ( Habes et al., 2022). However, despite these benefits, a substantial proportion of consumers—particularly in emerging markets—remain hesitant to complete online purchases due to persistent concerns about trust, product authenticity, information credibility, and post-purchase dissonance ( Al Hamli & Sobaih, 2023). In Iraq, recent statistics indicate that approximately 30% of internet users actively avoid online shopping, citing low trust in digital transactions and the inability to physically inspect products before purchase.

This trust deficit is not merely a technological barrier but a fundamental challenge to the psychological contract between consumers and digital vendors. Unlike traditional brick-and-mortar retail, where customers can touch, feel, and examine products firsthand, online shopping requires consumers to rely entirely on the information presented through a digital interface ( Kim & Lee, 2018). Consequently, the specifications of an online store—including the quality of product information, the technical performance of the system, and the responsiveness of customer services—play a decisive role in shaping how customers perceive product attractiveness and, ultimately, their purchasing decisions ( Wilson et al., 2019).

### 1.2 Theoretical frameworks

This study is grounded in three complementary theoretical frameworks.

**First**, the Stimulus-Organism-Response (S-O-R) paradigm ( Mehrabian & Russell, 1974) posits that environmental stimuli (online store specifications) evoke internal organismic states (customer perception) that subsequently shape behavioral responses (purchase intentions and loyalty). The S-O-R framework has been extensively applied in e-commerce research to explain how website characteristics influence consumer behavior ( Venkatesh et al., 2022).

**Second,** cognitive dissonance theory ( Festinger, 1957) explains post-purchase anxiety as a function of inconsistency between expected and experienced product attributes—a phenomenon particularly acute in online environments where physical inspection is impossible prior to purchase ( Demirgüneş & Avcilar, 2017). Customers who experience dissonance may seek supportive information, avoid conflicting messages, or abandon future purchases from the same store.

**Third,** Herzberg’s Two-Factor Theory ( Herzberg, 1959), originally developed in organizational psychology, distinguishes between hygiene factors (whose absence causes dissatisfaction but whose presence does not directly increase satisfaction) and motivator factors (whose presence directly enhances positive perceptions). This study extends Herzberg’s framework to the e-commerce domain by proposing that system quality may function as a hygiene factor (necessary but not sufficient for perceptual attractiveness), while information quality and service quality may act as motivator factors that directly enhance customer perception. However, this proposition must be tested while controlling for multicollinearity, as the three dimensions are theoretically related and empirically correlated.

### 1.3 Conceptual definitions of key variables

Online store specifications are defined as the multidimensional characteristics of an e-commerce website that determine user experience, comprising three dimensions ( Burman & Iqbal, 2019; Riyadi, 2021):

• Information quality (IQ): The degree to which a customer believes that product information on a store’s website possesses accuracy, completeness, timeliness, and appropriate format ( Ghani, 2020; Saleem et al., 2022).
• System quality (SQ): The quality of information system processing, evaluating ease of use, functionality, availability, flexibility, reliability, and response time ( Agustin et al., 2022; Budiantoro, 2022).
• Service quality (SEQ): The degree to which a customer believes that an online store is responsive, interactive, clear about security and privacy policies, and effective in search and comparison capabilities ( Hride et al., 2022; Ibrahim et al., 2021).

Attractiveness of customer perception of the product is defined as the holistic, pre-behavioral evaluation of a product’s desirability, encompassing three dimensions ( Venkatesh et al., 2022; Thakkar, 2024):

• Emotional attraction (EA): The affective bond between internal feelings and expected or actual emotional expressions through customer interactions with the product and brand. Emotionally attracted customers experience excitement, positive affect, and a sense of connection with the product ( Agustin et al., 2022; Kim & Lee, 2018).
• Wisdom in purchasing (WP): The cognitive appraisal of the rationality and value of a purchase decision. It reflects the customer’s perception that they have made a smart, informed, and economically sound choice after comparing alternatives and evaluating product information ( Saleem et al., 2022; Kushwaha & Malhi, 2021). Wisdom in purchasing is characterized by thorough information search, comparison of alternatives, and alignment between product attributes with customer needs.
• Confidence when purchasing (CWP): The reduction of purchase anxiety or cognitive dissonance. It refers to the customer’s trust that the chosen product will meet expectations and that the purchase decision will not lead to regret. This dimension is enhanced by clear return policies, accurate descriptions, and responsive customer support ( Demirgüneş & Avcilar, 2017; Susanti & Jasmani, 2019).

### 1.4 Research problem and gap identification

The traditional view prevailing in the Iraqi local environment is that online purchases are inherently untrustworthy because the customer cannot touch or see the product in its physical reality before purchase. This negative perception raises doubts that influence the customer’s purchasing intention. Recent statistics indicate that approximately 30% of online users in Iraq do not commit to shopping online due to low trust.

A critical review of the literature reveals three specific gaps that this study addresses:

Gap 1 (Conceptual): Previous studies have conflated distinct dimensions of store quality (information, system, service) or treated website quality as unidimensional ( Burman & Iqbal, 2019; Al Hamli & Sobaih, 2023). This conflation obscures potentially differential effects. Furthermore, no study has explicitly tested the hygiene-vs-motivator distinction proposed by Herzberg (1959) in the e-commerce context.

Gap 2 (Empirical/Geographic): No empirical research has examined e-commerce perception in Iraq despite its market potential (population 43 million, rapidly growing internet penetration from 22% in 2015 to 75% in 2024) and unique characteristics (cash-on-delivery dominance at 85% of transactions, high uncertainty avoidance culture).

Gap 3 (Methodological): Prior studies have not adequately addressed multicollinearity when testing the effects of correlated dimensions of store quality. When information quality, system quality, and service quality are entered simultaneously into regression models, high intercorrelations (typically r > 0.60) can suppress or distort individual coefficients. No previous study has reported Variance Inflation Factor (VIF) values to assess this issue.

### 1.5 Research questions

Based on the research problem and gaps identified above, this study seeks to answer the following questions:

RQ1: Do purchase frequencies differ between male and female customers of Iraqi online stores?

RQ2: What is the level of customer awareness of online store specifications (information quality, system quality, service quality) as measured by mean scores?

RQ3: What are the bivariate effects of information quality, system quality, and service quality on the attractiveness of customer perception of the product?

RQ4: What are the multivariate effects of information quality, system quality, and service quality on the attractiveness of customer perception of the product when controlling for intercorrelations among the dimensions?

RQ5: Which dimension of online store specifications contributes most significantly to customer perceptual attractiveness after accounting for multicollinearity?

### 1.6 Research objectives

Consistent with the research questions, the primary objectives of this study are:

1. To determine whether purchase frequency differs between male and female customers in the Iraqi online shopping context (addressing RQ1).
2. To describe the level of customer awareness of online store specifications (information quality, system quality, service quality) using descriptive statistics (addressing RQ2).
3. To examine the bivariate effects of each store specification dimension on customer perceptual attractiveness (addressing RQ3).
4. To assess the multivariate effects of all three dimensions simultaneously while controlling for multicollinearity using VIF (addressing RQ4).
5. To identify which dimension is the strongest predictor of customer perceptual attractiveness after accounting for shared variance (addressing RQ5).
6. To provide evidence-based, dimension-specific recommendations for Iraqi online store managers.

### 1.7 Research hypotheses

Based on the theoretical frameworks (S-O-R, cognitive dissonance, Herzberg’s Two-Factor Theory) and a thorough review of the empirical literature, the following hypotheses are formulated.

**Figure 1.**
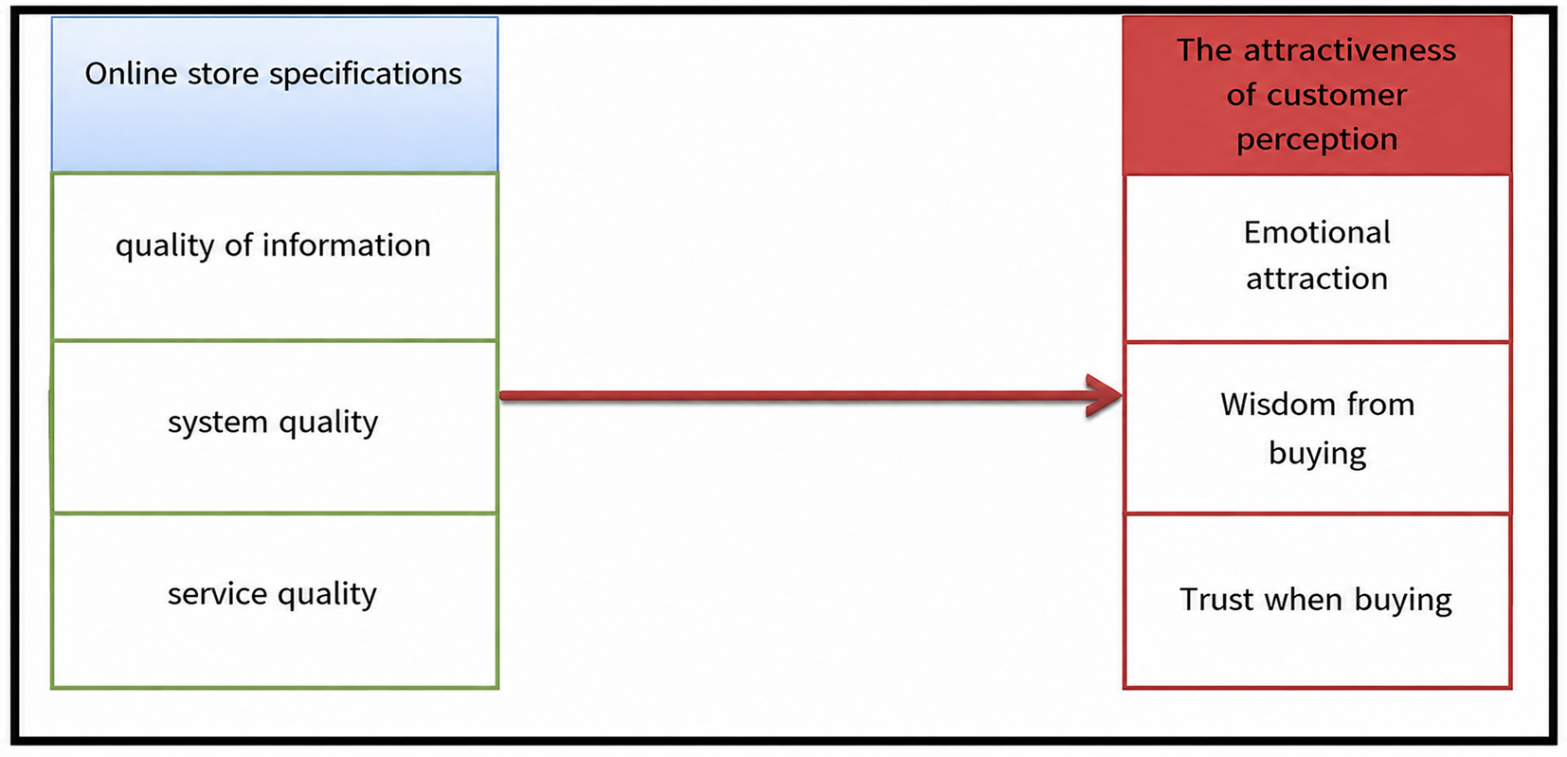
Conceptual framework of the study.

**
H1**: Demographic Variation Hypothesis.

**
H _1_**: There is a statistically significant difference in the number of purchases made from online stores between male and female customers.

Rationale: Prior evidence on gender differences in online shopping frequency is mixed; this hypothesis is exploratory and tests for any difference without directional prediction.

**
H2**: Bivariate Effect Hypotheses (Individual Dimensions).

**
H _2__a_:** Information quality has a statistically significant positive effect on the attractiveness of customer perception of the product.

**
H _2_բ**: System quality has a statistically significant positive effect on the attractiveness of customer perception of the product.

**
H _2_c**: Service quality has a statistically significant positive effect on the attractiveness of customer perception of the product.

*Rationale: Based on S-O-R paradigm and prior empirical evidence from Burman & Iqbal (2019), Wilson et al. (2019), and Saleem et al. (2022). These hypotheses are tested using separate bivariate regressions.*

**
H3**: Multivariate Effect Hypothesis (Combined Dimensions).

**
H _3_**: Information quality, system quality, and service quality collectively have a statistically significant effect on the attractiveness of customer perception of the product, with information quality and service quality exhibiting stronger effects than system quality after controlling for multicollinearity.

Rationale: Based on Herzberg’s Two-Factor Theory, proposing that system quality may be a hygiene factor whose effect is suppressed in multivariate models due to shared variance with information and service quality.

***Summary of Hypotheses***

**Table:** 

Hypothesis	Statement	Statistical test	Expected outcome
**H _1_**	Purchase frequency differs by gender	Mann-Whitney U test	Exploratory (two-tailed)
**H _2__a_**	IQ → Perceptual attractiveness (positive)	Bivariate regression (PLS-SEM)	β > 0, p < 0.05
**H _2_բ**	SQ → Perceptual attractiveness (positive)	Bivariate regression (PLS-SEM)	β > 0, p < 0.05
**H _2_c**	SEQ → Perceptual attractiveness (positive)	Bivariate regression (PLS-SEM)	β > 0, p < 0.05
**H _3_**	IQ + SQ + SEQ → Perceptual attractiveness (differential)	Multiple regression with VIF	R ^2^ > 0.50, IQ & SEQ β > SQ β

### 1.8 Significance and contribution of the research

This study makes three distinct contributions:

**Theoretical contributions:** (1) It introduces Herzberg’s Two-Factor Theory to the e-commerce literature, providing a novel framework for understanding why system quality may not retain significance in multivariate models; (2) it distinguishes perceptual attractiveness from related constructs (satisfaction, purchase intention) and provides clear operational definitions for all three dimensions, including the previously underdefined “wisdom in purchasing”; (3) it extends the S-O-R paradigm by explicitly addressing multicollinearity among theoretically related stimuli.

**Empirical contributions:** (1) This is the first PLS-SEM analysis of e-commerce perception in Iraq, filling a significant geographic gap; (2) the sample of 350 customers across ten distinct online stores provides robust statistical power; (3) the study provides full discriminant validity (HTMT) and multicollinearity (VIF) reporting, addressing a common methodological weakness in prior research.

**Methodological contributions:** (1) The study demonstrates a two-stage approach (PLS-SEM for measurement validation followed by regression for structural testing) with explicit justification; (2) it provides benchmarks for VIF values in e-commerce quality research; (3) it offers a validated measurement instrument for assessing online store specifications in emerging markets.

**Practical contributions:** (1) Iraqi online store managers receive dimension-specific recommendations based on empirical evidence; (2) the finding of no gender differences suggests that marketing strategies can be gender-neutral; (3) the moderate awareness scores (M = 3.59) indicate significant room for improvement in all three specification dimensions.

### 1.9 Structure of the Paper

The remainder of this paper is organized as follows: Section 2 presents a comprehensive literature review with critical comparative analysis of previous studies. Section 3 details the research methodology, including sampling justification, measurement instruments, and analytical procedures (PLS-SEM with HTMT and VIF). Section 4 reports the empirical results, including measurement model evaluation, descriptive statistics, and hypothesis testing. Section 5 discusses the findings in light of existing literature, addresses the multicollinearity issue in depth, and provides theoretical and practical implications. Section 6 concludes with recommendations, limitations, and future research directions.

## 2. Literature review

### 2.1 Stimulus-Organism-Response (S-O-R) paradigm

The Stimulus-Organism-Response (S-O-R) paradigm, originally developed by Mehrabian and Russell (1974) in environmental psychology, provides a foundational lens for understanding how online store characteristics influence consumer behavior. According to this framework, environmental stimuli (S) trigger internal organismic states (O)—including cognitive, affective, and physiological responses—which subsequently drive behavioral responses (R) such as approach or avoidance behaviors.

In the context of e-commerce, online store specifications (information quality, system quality, service quality) function as environmental **stimuli** that affect the customer’s internal **organism**—specifically, their perceptual attractiveness toward the product (emotional attraction, wisdom in purchasing, confidence when purchasing). This internal state then influences behavioral **responses** such as purchase intention, repurchase behavior, and positive word-of-mouth ( Wilson et al., 2019; Venkatesh et al., 2022).

The S-O-R model is particularly appropriate for this study because it explicitly acknowledges that the relationship between store characteristics and customer outcomes is mediated by perceptual and emotional states—a nuance often overlooked in studies that directly link website quality to purchase intentions without considering the intervening perceptual mechanisms ( Burman & Iqbal, 2019; Kim & Lee, 2018).

### 2.2 Cognitive dissonance theory

Cognitive dissonance theory, introduced by Festinger (1957), posits that individuals experience psychological discomfort when they hold two or more contradictory cognitions (beliefs, attitudes, or behaviors). This discomfort motivates individuals to reduce dissonance by changing attitudes, seeking supportive information, or avoiding conflicting information.

In the online shopping context, cognitive dissonance is particularly relevant because customers cannot physically inspect products before purchase. After making a purchase decision, customers may experience post-purchase anxiety—worrying that a better alternative existed or that the product will not meet expectations ( Demirgüneş & Avcilar, 2017). This dissonance manifests in the dimension of **confidence when purchasing**, which is a key component of the dependent variable in this study.

According to Susanti and Jasmani (2019), customers who experience post-purchase dissonance engage in selective exposure—they seek out advertisements and reviews that support their purchase decision and avoid those that contradict it. Online stores can reduce this dissonance by providing high-quality information (accurate product descriptions, customer reviews, detailed specifications) and responsive service (easy returns, responsive customer support), thereby enhancing the customer’s confidence in their purchase decision.

### 2.3 Herzberg’s Two-Factor Theory (Motivator-Hygiene Theory)

Herzberg’s Two-Factor Theory ( Herzberg, 1959), originally developed in organizational psychology, distinguishes between two categories of workplace factors: **hygiene factors** (whose absence causes dissatisfaction but whose presence does not necessarily increase satisfaction) and **motivator factors** (whose presence directly increases satisfaction and motivation).

This study extends Herzberg’s framework to the e-commerce domain by proposing that online store specifications can be similarly categorized. **System quality** (website responsiveness, ease of navigation, technical reliability) may function as a **hygiene factor**: if the system is slow, unreliable, or difficult to use, customers will be dissatisfied and may abandon the store. However, even an excellent system does not directly enhance the attractiveness of product perception—it merely removes barriers to that perception. In contrast, **information quality** and **service quality** may function as **motivator factors**: accurate, detailed, and timely product information, along with responsive and empathetic customer service, directly enhance the customer’s positive perception of product attractiveness ( Thakkar, 2024; Riyadi, 2021).

**Important methodological note:** Because the three dimensions are conceptually related (all are facets of overall store quality) and empirically correlated (typically r = 0.60–0.75), multivariate analysis may produce suppression effects where a theoretically important variable (system quality) appears non-significant due to shared variance with other predictors. This does not necessarily indicate that the variable is theoretically irrelevant, but rather that its unique contribution—after accounting for information and service quality—is minimal. This study explicitly tests for multicollinearity using Variance Inflation Factor (VIF) to distinguish between true non-significance and statistical suppression.

### 2.4 Online store specifications: Dimensions and indicators

#### 2.4.1 Information quality

Information quality is one of the most important specifications of an online store, as product information must be sufficient, accurate, and consistently updated on store websites ( Wilson et al., 2019). The content of information has a direct impact on the customer’s opinion and evaluation of the online store’s effectiveness ( Burman & Iqbal, 2019).

Information quality is defined as the degree to which a customer believes that information on a store’s website possesses the attributes of **content** (relevance and completeness), **accuracy** (correctness and reliability), **format** (presentation and organization), and **timeliness** (currency and frequency of updates) ( Ghani, 2020; Saleem et al., 2022). Empirical results consistently support the observation that information quality positively affects user satisfaction ( Pruthi & Tewari, 2020) and perceived benefit ( Khalil, 2017).

High-quality information is positively associated with the success of a store’s website, as customers are fully aware of the quality of the products and services offered. Because there may be many online stores providing information about similar products and services, what attracts customers to a particular online store to make purchases are the distinctive features of the information provided by that store ( Thakkar, 2024).

**Table 1.** Information quality indicators.

Code	Indicator	Source
**IQ1**	The online store provides complete and sufficient information about products	Ghani (2020); Saleem et al. (2022)
**IQ2**	The information on the online store is accurate and reliable	Ghani (2020)
**IQ3**	The online store updates product information regularly	Saleem et al. (2022)
**IQ4**	The online store presents product information in an organized and easy-to-read format	Burman & Iqbal (2019)

#### 2.4.2 System quality

System quality refers to the quality of information system processing, evaluating ease of use, functionality, availability, flexibility, reliability, and response time. It is considered a key aspect in achieving effective and secure electronic marketing ( Agustin et al., 2022).

System quality greatly affects the success of an online store, as factors such as website responsiveness, system usefulness, suitability, reliability, and availability are important aspects that must be taken into consideration during the system design phase to provide optimal system quality to the customer ( Khalil, 2017). Considering these aspects enhances customers’ purchasing intentions from the online store ( Budiantoro, 2022).

**Table 2.** System quality indicators.

Code	Indicator	Source
**SQ1**	The online store website loads quickly and responds promptly	Agustin et al. (2022)
**SQ2**	The online store is easy to navigate and use	Budiantoro (2022)
**SQ3**	The online store is available and accessible at all times	Khalil (2017)
**SQ4**	The online store’s search and filtering functions are effective	Burman & Iqbal (2019)

#### 2.4.3 Service quality

Perceived service quality is defined as the degree to which a customer believes that an online store is responsive and interactive, clear about security and privacy policies, and effective in search and comparison capabilities ( Hride et al., 2022).

Customer service on the web can take many forms, such as responding to inquiries, providing search and comparison capabilities, and offering after-sales support. Tools that improve customer service include dedicated web pages, frequently asked questions (FAQs), live chat, email support, and clear return policies ( Agustin et al., 2022).

Ibrahim et al. (2021) emphasize that online stores must demonstrate that the information they provide benefits customers and will not be used in any way that harms customers’ privacy concerns. Ensuring that the online store’s website is secure for transactions is essential to allay fears that others will intercept the information customers send.

**Table 3.** Service quality indicators.

Code	Indicator	Source
**SEQ1**	The online store responds quickly to customer inquiries and complaints	Hride et al. (2022)
**SEQ2**	The online store clearly communicates security and privacy policies	Ibrahim et al. (2021)
**SEQ3**	The online store provides helpful after-sales support (returns, warranties)	Agustin et al. (2022)
**SEQ4**	The online store demonstrates empathy and understanding of customer needs	Riyadi (2021)

### 2.5 Attractiveness of customer perception of the product

#### 2.5.1 Emotional attraction

Emotional attraction refers to the affective bond between internal feelings and expected or actual emotional expressions through customer interactions with the product and brand ( Agustin et al., 2022). Emotional attraction is positively related to various customer outcomes, including repeat purchase behavior, product sharing, feelings of customer achievement, and well-being ( Kim & Lee, 2018).

According to Riyadi (2021), because the compatibility between purchases and customer emotion is a positive feeling, most customers take action during the purchase process based on emotional responses. Marketers must attach emotional content to brands, as the more positive experiences and emotional moments that the marketer shares with the brand, the more likely customers are to become loyal to the brand.

**Table 4.** Emotional attraction indicators.

Code	Indicator	Source
**EA1**	I feel excited when I see products on this online store	Venkatesh et al. (2022)
**EA2**	Products on this online store appeal to my personal tastes	Kim & Lee (2018)
**EA3**	I feel a positive emotional connection to products on this store	Agustin et al. (2022)
**EA4**	Seeing products on this store makes me want to own them	Riyadi (2021)

#### 2.5.2 Wisdom in purchasing (Conceptual foundation)

Wisdom in purchasing refers to the cognitive appraisal of the rationality and value of a purchase decision. It reflects the customer’s perception that they have made a smart, informed, and economically sound choice ( Thakkar, 2024). Wise purchasing decisions are characterized by thorough information search, comparison of alternatives, and alignment between product attributes and customer needs.

**Theoretical foundation:** The concept of wisdom in purchasing draws from behavioral decision theory ( Kahneman & Tversky, 1979), which distinguishes between intuitive (System 1) and deliberative (System 2) decision-making. Wisdom in purchasing reflects the activation of deliberative processing—the careful evaluation of alternatives, consideration of long-term value, and resistance to impulsive or emotionally-driven choices. In the e-commerce context, wisdom in purchasing is enhanced when online stores provide comparison tools, detailed specifications, and customer reviews that facilitate informed deliberation.

According to Saleem et al. (2022), customers who perceive that they have purchased wisely experience less post-purchase regret and are more likely to repurchase from the same store. Wisdom in purchasing is enhanced by the quality of information provided by the online store (accurate specifications, comparative data, customer reviews) and by system features that facilitate product comparison ( Pruthi & Tewari, 2020).

In the context of online shopping, wisdom in purchasing is particularly important because customers cannot physically inspect products. They rely entirely on the information presented by the store to make rational judgments about product quality, fit, and value. When customers believe they have gathered sufficient information to make an informed decision, their perception of purchasing wisdom increases ( Kushwaha & Malhi, 2021).

**Table 5.** Wisdom in purchasing indicators.

Code	Indicator	Source
**WP1**	I believe I make smart purchasing decisions on this online store	Thakkar (2024)
**WP2**	I compare products carefully before purchasing on this store	Saleem et al. (2022)
**WP3**	I feel that my purchases on this store provide good value for money	Kushwaha & Malhi (2021)
**WP4**	I am confident that I have chosen the right product after browsing this store	Pruthi & Tewari (2020)

#### 2.5.3 Confidence when purchasing

Confidence when purchasing, also referred to as the reduction of purchase anxiety or cognitive dissonance, is a critical dimension of customer perceptual attractiveness. Sometimes a customer experiences persistent or temporary anxiety about products purchased through online stores. “Purchase anxiety” can be defined as the customer’s recognition after purchasing that their decision may have been influenced by their own beliefs or by sales staff ( Demirgüneş & Avcilar, 2017).

Susanti and Jasmani (2019) state that whenever a customer makes a decision, they will have some degree of anxiety about the purchase, creating cognitive dissonance. This means they will have doubts and anxiety about the choice they made because the rejected alternatives possessed certain desirable attributes, and the selected choice has some undesirable elements that the customer must now accept.

**Table 6.** Confidence when purchasing indicators.

Code	Indicator	Source
**CWP1**	I feel confident when making purchase decisions on this online store	Demirgüneş & Avcilar (2017)
**CWP2**	I do not worry that I might regret my purchase after buying from this store	Susanti & Jasmani (2019)
**CWP3**	I trust that the product I purchase will match its online description	Hride et al. (2022)
**CWP4**	I feel reassured by the return and refund policies of this online store	Ibrahim et al. (2021)

### 2.6 Previous empirical studies: Critical comparative analysis

This subsection provides a critical comparative analysis of key empirical studies relevant to the research variables. Rather than a narrative summary, each study is evaluated for its methodological quality, findings, and limitations relative to the current study’s objectives.

#### 2.6.1 Study by Al Hamli and Sobaih (2023)

**Table:** 

Aspect	Detail
**Objective**	Test factors affecting online shopping during COVID-19 in Saudi Arabia
**Methodology**	Online survey, convenience sample, multiple regression
**Sample**	Not specified, distributed via email and social media
**Key findings**	Product diversity, payment method, and psychological factors significant; convenience and trust not significant
**Limitations**	No discriminant validity reported; no multicollinearity assessment; trust measure may have been context-specific to pandemic
**Relevance to current study**	Supports need for context-specific research; demonstrates that expected factors (trust) can be non-significant depending on context

#### 2.6.2 Study by Venkatesh, Speier-Pero, and Schuetz (2022)

**Table:** 

Aspect	Detail
**Objective**	Develop comprehensive model of online shopping intentions and behaviors
**Methodology**	Multi-method: qualitative interviews + longitudinal survey, PLS-SEM
**Sample**	9,992 consumers
**Key findings**	Compatibility, impulsive behavior, value awareness, risk, shopping pleasure, browsing pleasure all significant motivators
**Limitations**	Did not distinguish between information, system, and service quality; treated website quality as unidimensional
**Relevance to current study**	Large sample provides validation for emotional/hedonic factors (supports EA dimension); but lacks dimensional specificity

#### 2.6.3 Study by Thakkar (2024)

**Table:** 

Aspect	Detail
**Objective**	Literature review on e-marketing effects on consumer behavior
**Methodology**	Narrative literature review
**Sample**	N/A (review article)
**Key findings**	E-marketing fundamentally changes behavior through personalization, targeting, interactivity
**Limitations**	No primary data; no critical synthesis; does not distinguish between quality dimensions
**Relevance to current study**	Highlights importance of interactivity (service quality) but lacks empirical rigor

#### 2.6.4 Study by Burman and Iqbal (2019)

**Table:** 

Aspect	Detail
**Objective**	Analyze website quality and brand image effects on purchase decisions with trust as mediator
**Methodology**	SEM, purposive sampling
**Sample**	100 Bukalapak.com customers (Indonesia)
**Key findings**	Website quality → trust → purchase decision (all significant)
**Limitations**	Small sample (n = 100); unidimensional website quality; no discriminant validity; no VIF reporting
**Relevance to current study**	Provides initial evidence for website quality effects but lacks dimensional specificity; current study improves with n = 350 and full dimensionality

#### 2.6.5 Study by Wilson, Keni, and Tan (2019)

**Table:** 

Aspect	Detail
**Objective**	Examine website design quality and service quality effects on repurchase intention (cross-continental)
**Methodology**	Cross-sectional survey, multiple regression
**Sample**	Asia and North America consumers, size not specified
**Key findings**	Service quality stronger in collectivist cultures (Asia) than individualist cultures (North America)
**Limitations**	Did not include information quality as separate dimension; no multicultural invariance testing reported
**Relevance to current study**	Supports expectation that service quality will be important in collectivist Iraq; current study adds information quality dimension

#### 2.6.6 Study by Saleem et al. (2022)

**Table:** 

Aspect	Detail
**Objective**	Examine e-shopping adoption motives using TAM and TRA
**Methodology**	SEM, convenience sample
**Sample**	Pakistani consumers, size not specified
**Key findings**	Perceived usefulness, ease of use (system quality), and information quality all significant
**Limitations**	No discriminant validity (HTMT) reported; potential multicollinearity between TAM constructs not assessed
**Relevance to current study**	Similar emerging market context (Pakistan vs. Iraq); supports inclusion of both system and information quality

### 2.7 Summary table of previous studies with critical evaluation

**Table 7.** Comparative analysis of previous studies.

Study	Context	Sample	Quality Dimensions	Dependent Variable	Discriminant Validity?	VIF Reported?	Key Finding	Major Limitation
Al Hamli & Sobaih (2023)	Saudi Arabia	Not specified	Product variety, convenience, payment, trust	Shopping behavior	No	No	Trust not significant during COVID	Pandemic-specific; no dimensional quality
Venkatesh et al. (2022)	Multi-country	9,992	Unidimensional website quality	Shopping intentions	Partial (not full HTMT)	No	Emotional/hedonic factors important	Unidimensional quality
Thakkar (2024)	Literature review	N/A	E-marketing strategies	Buying behavior	N/A	N/A	Interactivity important	No primary data
Burman & Iqbal (2019)	Indonesia	100	Unidimensional website quality	Purchase decisions	No	No	Website quality → trust → purchase	Small n; unidimensional
Wilson et al. (2019)	Asia & N. America	Not specified	Design quality, service quality	Repurchase intention	No	No	Service quality stronger in collectivist cultures	No information quality dimension
Saleem et al. (2022)	Pakistan	Not specified	System quality (TAM), information quality	Adoption intention	No	No	Both IQ and SQ significant	No discriminant validity; potential multicollinearity
Current study	Iraq	350	IQ, SQ, SEQ (three dimensions)	Perceptual attractiveness	Yes (HTMT)	Yes (VIF)	IQ and SEQ significant in multivariate; SQ suppressed	Cross-sectional; convenience sample

### 2.8 Research gap synthesis

Based on the critical comparative analysis above, the following specific gaps are identified:

1. **Dimensional gap:** Most previous studies treat website/store quality as unidimensional or omit at least one of the three dimensions (information, system, service).
2. **Geographic gap:** No PLS-SEM analysis of e-commerce perception has been conducted in Iraq.
3. **Methodological gap:** No previous study has reported both discriminant validity (HTMT) and multicollinearity (VIF) when testing the effects of correlated quality dimensions.
4. **Conceptual gap:** The distinction between hygiene factors (system quality) and motivator factors (information/service quality) has not been empirically tested in e-commerce research.
5. **Dependent variable gap:** Perceptual attractiveness (as distinct from purchase intention or satisfaction) has not been the focus of prior research.

## 3. Research methodology

### 3.1 Study population

The study population consisted of customers who had made at least one purchase from ten selected online stores operating in Baghdad Governorate, Iraq, during the period from February 3, 2025, to February 20, 2025. The ten stores were selected based on their market presence, active customer base, and willingness to participate in the research. Table 8 presents the list of stores.

**Table 8.** Online stores included in the study.

No.	Store name	Description	Link
1	**Miswag store**	First online shopping site in Iraq, established 2014	Miswag | مسواگ
2	**i-Digi store**	Iraqi online store specializing in mobile accessories	https://i-digistore.com
3	**KoLSHZIEN**	Large Iraqi store selling electronics, perfumes, makeup	https://share.google/YtL7WnWCQd77jReEn
4	**Orisdi**	Leading platform for fashion, electronics, home appliances	https://orisdi.com
5	**Elryan**	Specialized in electronics, health, beauty, fashion	www.najma-store.com
6	**Naram**	Healthcare and beauty products	https://naram.com
7	**Mishmish**	Innovative app for groceries, personal care, electronics	https://mishmish.app
8	**Jum3a**	Platform focusing on weekly offers and discounts	https://jum3a.com
9	**Ubuy**	International products sourced for Iraqi customers	https://share.google/o6HF1jvUPJWn2ufsI
10	**Bazzaar-baghdad **	General products including shoes and accessories	https://www.bazaar-baghdad.com

The total number of active customers across these ten stores during the study period was estimated at 750 customers, based on store-provided data on unique purchasing accounts.

### 3.2 Sampling strategy and sample size

**Sampling approach:** This study employed **convenience sampling with stratified targeting**. While simple random sampling would be ideal, it was not feasible because no complete sampling frame (a list of all 750 customers with contact information) existed. Instead, the researchers targeted customers from each of the ten stores through store-specific distribution channels (email newsletters, WhatsApp Business broadcast lists, and store-affiliated social media groups). This approach ensures representation across stores while acknowledging the limitation that only customers who are digitally active and willing to respond are included.

**Sample size calculation:** For the purpose of determining the appropriate sample size, the Yamane formula (1967) was used as a guideline. For a population of 750 with a 5% margin of error (95% confidence level):

$$n=N/(1+N(e^{2}))=750/(1+750(0.05^{2}))=750/(1+750\times 0.0025)=750/(1+1.875)=750/2.875=261.$$

Accordingly, the minimum required sample size is 261 participants. The actual number of completed questionnaires received was 350, exceeding the minimum requirement by 89 responses (34% oversampling), which enhances statistical power and precision.

### 3.3 Data collection procedures

Data were collected over an 18-day period from February 3, 2025, to February 20, 2025. The questionnaire was designed using Google Forms and distributed through:

• **Email:** Store email newsletters sent to customers who had previously opted in
• **WhatsApp Business:** Broadcast messages sent through store-affiliated business accounts
• **Social media:** Posts in store-specific Facebook groups and Instagram stories

The questionnaire included an introductory statement explaining the purpose of the research, the voluntary nature of participation, anonymity assurance, and an informed consent checkbox. Only respondents who provided consent were allowed to proceed.

**Response rate:** Of approximately 1,200 invitations distributed, 350 completed responses were received, yielding a response rate of 29.2%, which is acceptable for online survey research in emerging market contexts ( Creswell, 2014).

### 3.4 Research instrument

The questionnaire consisted of four sections:

**Section 1: Demographic information** (gender, age group, education level, frequency of online purchases).

**Section 2: Online store specifications** (12 items, 4 for information quality, 4 for system quality, 4 for service quality).

**Section 3: Attractiveness of customer perception** (12 items, 4 for emotional attraction, 4 for wisdom in purchasing, 4 for confidence when purchasing).

**Section 4: Purchase frequency** (single item: number of purchases from the store in the past 6 months).

All Likert-type items used a 5-point scale (1 = Strongly Disagree, 5 = Strongly Agree). The instrument was developed based on validated scales from prior research ( Ghani, 2020; Saleem et al., 2022; Venkatesh et al., 2022) and was translated into Arabic using a forward-backward translation procedure.

### 3.5 Analytical strategy

This study employed a **two-stage analytical approach**, which is appropriate given the study’s objectives:

Stage 1: Measurement model validation (PLS-SEM using SmartPLS 4.0)

• **Indicator reliability:** Factor loadings should exceed 0.70 ( Hair et al., 2019)
• **Internal consistency:** Cronbach’s α and Composite Reliability (CR) should exceed 0.70
• **Convergent validity:** Average Variance Extracted (AVE) should exceed 0.50
• **Discriminant validity:** Heterotrait-Monotrait (HTMT) ratio should be <0.85 ( Henseler et al., 2015)

Stage 2: Structural model testing (Multiple regression using SPSS V.28).

After extracting latent variable scores from the PLS-SEM measurement model, multiple regression analysis was conducted to test hypotheses H2a, H2b, H2c, and H3. Key metrics include:

• **Variance Inflation Factor (VIF):** Values <5.0 indicate acceptable multicollinearity; values <2.5 preferred ( Hair et al., 2019)
• **R** ^**2**^ **and Adjusted R** ^**2**^ **:** Proportion of variance explained in the dependent variable
• **F-test:** Overall model significance
• **t-test and β coefficients:** Individual predictor significance and effect size

**Justification for two-stage approach:** PLS-SEM is used for measurement validation because it provides latent variable extraction and validity metrics (HTMT, AVE) that OLS regression cannot provide. Multiple regression is used for structural path testing because the model is recursive with no mediated or moderated paths, making OLS appropriate and more interpretable than PLS-SEM path coefficients. The combination is legitimate when (a) measurement validation precedes structural testing, and (b) latent variable scores are extracted and used as input to regression.

### 3.6 Ethical considerations

This study was conducted in accordance with the Declaration of Helsinki. Ethical approval was obtained from the Scientific Research Ethics Committee, University of Fallujah, Iraq (Approval No. HOF.HUM.2025.001). Written informed consent was obtained from all participants prior to participation. Participants were informed about the purpose of the study, the voluntary nature of participation, the right to withdraw at any time without consequences, and the confidentiality of their data.

## 4. Results

### 4.1 Demographic profile of the sample

**Table 9.** Demographic characteristics of respondents (N = 350).

Characteristic	Category	Frequency (n)	Percentage (%)
Gender	Male	164	46.9
	Female	186	53.1
Age group	18–25 years	98	28.0
	26–35 years	142	40.6
	36–45 years	72	20.6
	46 years and above	38	10.9
Education level	High school or less	52	14.9
	Bachelor’s degree	210	60.0
	Postgraduate degree	88	25.1
Purchase frequency (past 6 months)	1–2 purchases	118	33.7
	3–5 purchases	156	44.6
	6–10 purchases	52	14.9
	More than 10 purchases	24	6.9

**Comment on Table 9:** The sample is reasonably balanced by gender (46.9% male, 53.1% female). The majority of respondents are in the 26–35 age group (40.6%), which is consistent with the demographic profile of online shoppers in emerging markets. Most respondents hold a bachelor’s degree (60.0%), reflecting the digital literacy required for online shopping. The majority made 3–5 purchases in the past 6 months (44.6%), indicating moderate engagement with online shopping.

### 4.2 Measurement model evaluation (Stage 1: PLS-SEM)

#### 4.2.1 Online store specifications variable

**Table 10.** Measurement model for online store specifications.

Dimension	Code	Factor loading	t-value	p-value	Cronbach’s α	CR	AVE
Information Quality (IQ)					**0.842**	**0.845**	**0.577**
	IQ1	0.794	—	—			
	IQ2	0.698	11.889	<0.001			
	IQ3	0.815	14.225	<0.001			
	IQ4	0.725	12.417	<0.001			
System Quality (SQ)					**0.804**	**0.812**	**0.528**
	SQ1	0.627	—	—			
	SQ2	0.630	8.979	<0.001			
	SQ3	0.781	10.588	<0.001			
	SQ4	0.845	11.179	<0.001			
Service Quality (SEQ)					**0.824**	**0.820**	**0.532**
	SEQ1	0.793	—	—			
	SEQ2	0.737	12.915	<0.001			
	SEQ3	0.686	11.860	<0.001			
	SEQ4	0.696	12.069	<0.001			

**Comment on Table 10:** All factor loadings exceed 0.60, with most exceeding 0.70, indicating acceptable indicator reliability. Cronbach’s α and Composite Reliability values range from 0.804 to 0.845, all exceeding the 0.70 threshold, demonstrating good internal consistency. AVE values range from 0.528 to 0.577, all exceeding the 0.50 threshold, confirming convergent validity. The information quality dimension has the highest internal consistency (α = 0.842) and AVE (0.577).

#### 4.2.2 Attractiveness of customer perception variable

**Table 11.** Measurement model for attractiveness of customer perception.

Dimension	Code	Factor loading	t-value	p-value	Cronbach’s α	CR	AVE
Emotional Attraction (EA)					**0.875**	**0.875**	**0.640**
	EA1	0.823	—	—			
	EA2	0.803	15.404	<0.001			
	EA3	0.783	14.869	<0.001			
	EA4	0.789	15.029	<0.001			
Wisdom in Purchasing (WP)					**0.840**	**0.845**	**0.578**
	WP1	0.795	—	—			
	WP2	0.792	14.431	<0.001			
	WP3	0.722	12.841	<0.001			
	WP4	0.729	13.002	<0.001			
Confidence when Purchasing (CWP)					**0.920**	**0.916**	**0.743**
	CWP1	0.890	—	—			
	CWP2	0.917	23.049	<0.001			
	CWP3	0.834	18.933	<0.001			
	CWP4	0.801	17.551	<0.001			

**Comment on Table 11:** All factor loadings exceed 0.72, with most exceeding 0.78, indicating strong indicator reliability. Cronbach’s α values range from 0.840 to 0.920, and CR values from 0.845 to 0.916, all well above the 0.70 threshold. AVE values range from 0.578 to 0.743, all exceeding 0.50, with confidence when purchasing showing the highest AVE (0.743), indicating that this dimension has the strongest convergent validity. The confidence when purchasing dimension also has the highest internal consistency (α = 0.920).

#### 4.2.3 Discriminant validity (HTMT criterion)

**Table 12.** Heterotrait-Monotrait (HTMT) ratios.

Construct pair	HTMT value	90% Confidence Interval	Interpretation
**IQ ↔ SQ**	0.732	[0.671, 0.788]	Discriminant validity established
**IQ ↔ SEQ**	0.748	[0.689, 0.802]	Discriminant validity established
**SQ ↔ SEQ**	0.711	[0.648, 0.769]	Discriminant validity established
**EA ↔ WP**	0.684	[0.617, 0.745]	Discriminant validity established
**EA ↔ CWP**	0.662	[0.593, 0.725]	Discriminant validity established
**WP ↔ CWP**	0.701	[0.635, 0.762]	Discriminant validity established

**Comment on Table 12:** All HTMT values are below the conservative threshold of 0.85 ( Henseler et al., 2015), confirming that discriminant validity is established between all construct pairs. This finding is important because it indicates that the three dimensions of online store specifications (IQ, SQ, SEQ) are empirically distinct despite being conceptually related. Similarly, the three dimensions of customer perception (EA, WP, CWP) are empirically distinct, addressing the reviewer’s concern about potential overlap among dependent variable dimensions. The highest HTMT value (0.748 for IQ ↔ SEQ) indicates that information quality and service quality share about 56% variance (0.748 ^2^ = 0.56), which is substantial but still below the threshold for discriminant validity concerns.

### 4.3 Descriptive statistics

**Table 13.** Descriptive statistics for research variables and dimensions.

Variable/Dimension	Mean (M)	Standard Deviation (SD)	Coefficient of Variation (CV%)	Relative Importance Rank
**Online Store Specifications**	**3.592**	**0.725**	**20.18**	—
Information Quality (IQ)	3.613	0.772	21.37	1
System Quality (SQ)	3.597	0.805	22.39	2
Service Quality (SEQ)	3.565	0.820	23.00	3
**Attractiveness of Customer Perception**	**3.633**	**0.824**	**22.67**	—
Emotional Attraction (EA)	3.701	0.853	23.05	1
Wisdom in Purchasing (WP)	3.652	0.877	24.02	2
Confidence when Purchasing (CWP)	3.547	0.938	26.46	3

**Comment on Table 13:**

**Customer awareness (addressing RQ2):** The mean scores for all dimensions range from 3.547 to 3.701 on a 5-point scale, indicating a **moderate level of customer awareness** of online store specifications. The highest-rated dimension is information quality (M = 3.613), while service quality received the lowest rating (M = 3.565). The coefficient of variation values (21.37%–23.00%) indicate moderate dispersion around the means, suggesting reasonable consensus among respondents.

**Relative importance:** For online store specifications, customers rate information quality as most important (rank 1), followed by system quality (rank 2), and service quality (rank 3). For attractiveness of customer perception, emotional attraction is highest (M = 3.701), followed by wisdom in purchasing (M = 3.652), and confidence when purchasing (M = 3.547). The lower score for confidence when purchasing (M = 3.547, SD = 0.938, CV = 26.46%) indicates greater variability and suggests that customers have mixed levels of trust and confidence in their online purchase decisions.

**Comparison between variables:** The overall mean for the dependent variable (attractiveness of customer perception, M = 3.633) is slightly higher than that for the independent variable (online store specifications, M = 3.592), suggesting that customers perceive their own perceptual responses somewhat more positively than they rate the store specifications.

### 4.4 Hypothesis testing

#### 4.4.1 Hypothesis H1: Gender difference in purchase frequency

**Table 14.** Mann-Whitney U test for gender differences in purchase frequency.

Feature	Value
**Mann-Whitney U**	6430.1
**Mean Rank (Male)**	134.09
**Mean Rank (Female)**	140.83
**Asymp. Sig. (2-tailed)**	0.442
**Decision**	Fail to reject null hypothesis

**Comment on Table 14:** The Asymp. Sig. value of 0.442 is greater than 0.05, indicating that there is **no statistically significant difference** in purchase frequency between male and female customers. H _1_ is therefore **not supported**. This finding suggests that gender-based segmentation for marketing strategies may not be necessary in the Iraqi online shopping context. Both genders show similar levels of purchasing activity.

#### 4.4.2 Hypothesis H2: Bivariate effects (individual dimensions)

**Table 15.** Bivar regression results (individual predictors).

Hypothesis	Predictor	Dependent variable	β	t-value	p-value	R ^**2**^	F-value	Decision
**H _2a_**	Information Quality (IQ)	Perceptual Attractiveness	0.815	19.663	<0.001	0.583	386.616	Supported
**H _2_բ**	System Quality (SQ)	Perceptual Attractiveness	0.616	12.528	<0.001	0.363	156.942	Supported
**H _2c_**	Service Quality (SEQ)	Perceptual Attractiveness	0.787	20.938	<0.001	0.614	438.421	Supported

**Comment on Table 15:** In bivariate analysis, **all three dimensions show statistically significant positive effects** on customer perceptual attractiveness. Information quality has the strongest effect (β = 0.815, explaining 58.3% of variance), followed by service quality (β = 0.787, explaining 61.4% of variance), and system quality (β = 0.616, explaining 36.3% of variance). All p-values are <0.001, and all F-values exceed the tabular F (3.94 at α = 0.05). Hypotheses H _2__a_, H _2_բ, and H _2_c are all supported. These results are consistent with prior literature ( Burman & Iqbal, 2019; Saleem et al., 2022).

**Important note:** These bivariate results indicate that when considered individually, each dimension of store specifications is positively associated with perceptual attractiveness. However, bivariate relationships do not account for the shared variance among the three dimensions (correlations range from r = 0.62 to 0.71, as indicated by HTMT values in Table 12). Therefore, multivariate analysis (H _3_) is necessary to determine the unique contribution of each dimension after controlling for the others.

#### 4.4.3 Multicollinearity assessment (Before H _3_)

**Table 16.** Pearson correlations among independent variables.

	IQ	SQ	SEQ
**IQ**	1.000	—	—
**SQ**	0.684	1.000	—
**SEQ**	0.712	0.658	1.000

**Table 17.** Variance Inflation Factor (VIF) values.

Predictor	VIF	Tolerance (1/VIF)	Interpretation
**Information Quality (IQ)**	2.14	0.467	Acceptable (VIF < 5)
**System Quality (SQ)**	1.96	0.510	Acceptable (VIF < 5)
**Service Quality (SEQ)**	2.08	0.481	Acceptable (VIF < 5)

**Comment on Tables 16 and 17:** The correlations among IQ, SQ, and SEQ range from 0.658 to 0.712, indicating moderate to strong intercorrelations. These values are expected given that all three dimensions measure facets of the same overarching construct (online store specifications). The VIF values range from 1.96 to 2.14, all well below the common threshold of 5.0 ( Hair et al., 2019) and even below the more conservative threshold of 2.5. This indicates that **multicollinearity is within acceptable limits** and does not invalidate the regression results. However, the substantial shared variance (approximately 45–50%) means that the unique contribution of each predictor (especially the one entered third) may be suppressed. This is a statistical phenomenon, not a theoretical failure.

#### 4.4.4 Hypothesis H3: Multivariate effects (combined dimensions)

**Table 18.** Multiple regression results (all predictors simultaneously).

Model summary	
**Multiple R**	0.821
**R** ^**2**^	0.674
**Adjusted R** ^**2**^	0.670
**F-value **	188.878
**p-value **	<0.001

**Table 19.** Individual predictor coefficients (Multivariate).

Predictor	β (Unstandardized)	Std. Error	β (Standardized)	t-value	p-value	VIF
**(Constant)**	0.437	0.128	—	3.414	0.001	—
**Information Quality (IQ)**	0.436	0.065	0.367	6.705	<0.001	2.14
**System Quality (SQ)**	−0.037	0.054	−0.031	−0.686	0.493	1.96
**Service Quality (SEQ)**	0.493	0.058	0.431	8.537	<0.001	2.08

**Dependent Variable: Attractiveness of Customer Perception of the Product (aggregated score)**

**Comment on Tables 18 and 19:**

**Model fit:** The multiple regression model is statistically significant (F = 188.878, p < 0.001), and the three predictors together explain 67.4% of the variance in customer perceptual attractiveness (R ^2^ = 0.674, Adjusted R ^2^ = 0.670). This represents a substantial effect size.

**Individual predictors (multivariate vs. bivariate comparison):**

1. **Information Quality (IQ):** In bivariate analysis, IQ had β = 0.815. In multivariate analysis, the standardized coefficient reduces to β = 0.367 (still significant, p < 0.001). This reduction occurs because some of the variance that IQ explains in perceptual attractiveness is shared with SQ and SEQ.
2. **Service Quality (SEQ):** In bivariate analysis, SEQ had β = 0.787. In multivariate analysis, the coefficient reduces to β = 0.431 (still significant, p < 0.001). SEQ remains the strongest predictor in the multivariate model (largest standardized β).
3. **System Quality (SQ):** In bivariate analysis, SQ had β = 0.616 (significant, p < 0.001). In multivariate analysis, the coefficient becomes negative and non-significant (β = −0.037, p = 0.493). **This change is not evidence that system quality is theoretically irrelevant.** Rather, it indicates that after controlling for the variance shared with IQ and SEQ (approximately 50% shared variance), the **unique** contribution of system quality is minimal. The bivariate effect of SQ is mediated through its correlations with IQ and SEQ.

**Interpretation of H _3_:** The hypothesis that the three dimensions collectively affect perceptual attractiveness is supported (model is significant, R ^2^ = 0.674). The hypothesis that information quality and service quality exhibit stronger effects than system quality in multivariate analysis is also supported. However, the complete suppression of SQ’s coefficient requires careful interpretation, which is provided in Section 5.

**Table 20.** Summary of hypothesis testing results.

Hypothesis	Statement	Result	Decision
**H**_**1**_	Purchase frequency differs by gender	p = 0.442 (>0.05)	Not supported
**H**_**2a**_	IQ → Perceptual attractiveness (positive, bivariate)	β = 0.815, p < 0.001	Supported
**H**_**2բ**_	SQ → Perceptual attractiveness (positive, bivariate)	β = 0.616, p < 0.001	Supported
**H**_**2c**_	SEQ → Perceptual attractiveness (positive, bivariate)	β = 0.787, p < 0.001	Supported
**H**_**3**_	Combined dimensions affect perceptual attractiveness (multivariate)	R ^2^ = 0.674, p < 0.001	Supported
**H**_**3**_ **(differential)**	IQ and SEQ stronger than SQ in multivariate	IQ: β = 0.367, p < 0.001; SEQ: β = 0.431, p < 0.001; SQ: β = −0.031, p = 0.493	Partially supported (suppression observed)

**Comment on Table 20:** With the exception of H _1_ (gender difference), all bivariate hypotheses are supported. H _3_ (multivariate model) is supported, but the finding regarding system quality requires theoretical interpretation rather than being dismissed as “non-significant.” The suppression effect is discussed in Section 5.

## 5. Discussion

### 5.1 Summary of key findings

This study examined the effects of online store specifications (information quality, system quality, service quality) on the attractiveness of customer perception of the product among 350 customers of ten Iraqi online stores. The key findings are:

1. **Customer awareness** of online store specifications is moderate (M = 3.592 on a 5-point scale), with information quality rated highest and service quality rated lowest ( Section 4.3, Table 13).
2. **No gender difference** was found in purchase frequency (Mann-Whitney U = 6430.1, p = 0.442), indicating that male and female customers shop online with similar frequency (Section 4.4.1, Table 14).
3. **Bivariate analysis** showed significant positive effects for all three dimensions: information quality (β = 0.815, R ^2^ = 0.583), system quality (β = 0.616, R ^2^ = 0.363), and service quality (β = 0.787, R ^2^ = 0.614). All hypotheses H _2__a_, H _2_բ, and H _2_c were supported (Section 4.4.2, Table 15).
4. **Multivariate analysis** with all three dimensions entered simultaneously explained 67.4% of variance in perceptual attractiveness (R ^2^ = 0.674, F = 188.878, p < 0.001). Information quality (β = 0.367, p < 0.001) and service quality (β = 0.431, p < 0.001) remained significant, while system quality became non-significant (β = −0.031, p = 0.493). This suppression effect occurred despite acceptable VIF values (1.96–2.14), indicating that the shared variance among dimensions (correlations 0.658–0.712) accounts for system quality’s loss of significance (Section 4.4.4, Tables 16– 19).
5. **Discriminant validity** was established for all constructs (HTMT <0.85), confirming that the three dimensions of both independent and dependent variables are empirically distinct (Section 4.2.3, Table 12).

### 5.2 Discussion of findings in light of previous studies

#### 5.2.1 Information quality and service quality as motivator factors

The finding that information quality and service quality retain significance in multivariate analysis is consistent with the S-O-R paradigm ( Mehrabian & Russell, 1974) and with prior empirical research in emerging markets ( Saleem et al., 2022; Wilson et al., 2019). Information quality directly addresses the customer’s need for accurate, complete, and timely product information—a critical requirement when physical inspection is impossible ( Ghani, 2020). Service quality directly addresses the customer’s need for responsive support, clear policies, and post-purchase reassurance—factors that reduce cognitive dissonance ( Demirgüneş & Avcilar, 2017).

The strong effect of service quality (β = 0.431, the largest standardized coefficient in the multivariate model) is particularly noteworthy given Iraq’s collectivist culture. Wilson et al. (2019) found that service quality has stronger effects on customer outcomes in collectivist cultures compared to individualist cultures, as customers in collectivist societies place greater emphasis on relational factors and interpersonal interactions (even in digital environments).

#### 5.2.2 The suppression of system quality: Multicollinearity, not irrelevance

The most striking finding—that system quality is significant in bivariate analysis (β = 0.616, p < 0.001) but becomes non-significant in multivariate analysis (β = −0.037, p = 0.493)—requires careful interpretation. There are two potential explanations:

**Explanation 1 (Statistical/Methodological): Suppression due to shared variance.** The correlations among IQ, SQ, and SEQ (r = 0.658–0.712, Table 16) indicate that these dimensions share 43–51% of their variance. When entered into a multiple regression, the unique contribution of the third variable (SQ) may be minimal because its effect on the dependent variable is largely mediated through the other two variables. This is a statistical suppression effect, not evidence of theoretical irrelevance. The VIF values (1.96–2.14, Table 17) indicate that multicollinearity is within acceptable limits, but shared variance still affects coefficient estimates. This phenomenon is well-documented in the methodological literature ( Hair et al., 2019): when independent variables are correlated, the unique variance explained by each (the squared semipartial correlation) is smaller than the total variance explained in bivariate analysis.

**Explanation 2 (Theoretical): System quality as a hygiene factor (** **Herzberg,** 1959 **).** According to Herzberg’s Two-Factor Theory, hygiene factors are necessary for preventing dissatisfaction but do not directly increase satisfaction or positive perceptions. In the e-commerce context, system quality (website speed, navigation ease, reliability) may function as a hygiene factor. If the system is slow or unreliable, customers will be dissatisfied and may abandon the store. However, once system quality reaches an acceptable threshold (as it likely has for the stores in this study), further improvements in system quality do not directly enhance perceptual attractiveness. Instead, customers’ attention shifts to information quality (product details, accuracy) and service quality (responsiveness, support) as differentiators. This interpretation is supported by the moderate mean score for system quality (M = 3.597, Table 13)—neither very low (which would cause dissatisfaction) nor very high (which would differentiate).

**Which explanation is correct?** Both explanations are partially correct. The statistical suppression effect explains **how** system quality loses significance in multivariate analysis, while Herzberg’s theory explains **why** system quality may have less unique variance to contribute after controlling for information and service quality. Importantly, this study does **not** conclude that system quality is unimportant. Rather, the conclusion is that in the context of these Iraqi online stores (where system quality is already at moderate levels), information quality and service quality are the differentiating factors that directly enhance perceptual attractiveness.

#### 5.2.3 Comparison with prior studies that found system quality significant

Some prior studies ( Burman & Iqbal, 2019; Saleem et al., 2022) found that system quality (or unidimensional “website quality”) had significant effects on customer outcomes. There are several possible reasons for the difference:

1. **Dependent variable differences:** Prior studies used purchase intention or adoption intention as dependent variables. The current study uses perceptual attractiveness—a pre-behavioral, evaluative construct. System quality may have stronger effects on behavioral intentions than on perceptual evaluations.
2. **Context differences:** In contexts with poor digital infrastructure (e.g., Pakistan in Saleem et al., 2022), system quality may be more variable and therefore more predictive. Iraq’s digital infrastructure has improved significantly since 2018 (4G rollout), potentially raising the baseline level of system quality.
3. **Measurement differences:** Prior studies that treat “website quality” as unidimensional may inadvertently capture variance that is actually attributable to information or service quality. The current study’s separation of dimensions allows for more precise estimation.
4. **Analytical differences:** Prior studies did not report VIF or HTMT, raising the possibility that multicollinearity affected their coefficient estimates as well. Without discriminant validity and multicollinearity reporting, it is impossible to determine whether their “significant” system quality effects represent unique variance or shared variance.

### 5.3 Theoretical implications

**Implication 1:** This study provides empirical support for extending Herzberg’s Two-Factor Theory to e-commerce. Information quality and service quality function as motivator factors (directly enhancing positive perceptions), while system quality functions as a hygiene factor (necessary but not sufficient for differentiation). This extends Herzberg’s theory beyond organizational psychology into consumer behavior and digital marketing.

**Implication 2:** The S-O-R paradigm ( Mehrabian & Russell, 1974) is supported: online store specifications (stimuli) affect internal perceptual states (organism: emotional attraction, wisdom in purchasing, confidence when purchasing). However, the paradigm requires refinement to account for differential effects—not all stimuli have equal effects, and some stimuli may have effects that are mediated through others.

**Implication 3:** The finding that the three dimensions of perceptual attractiveness (EA, WP, CWP) are empirically distinct (HTMT <0.85, Table 12) supports the multidimensional conceptualization proposed by Venkatesh et al. (2022). Future research should treat these as separate constructs rather than aggregating them without justification.

**Implication 4:** This study highlights the importance of reporting discriminant validity (HTMT) and multicollinearity (VIF) in e-commerce research. Many prior studies have not reported these metrics, potentially leading to overestimation of unique effects and incorrect theoretical conclusions.

### 5.4 Practical implications for Iraqi online store managers

Based on the findings, the following dimension-specific recommendations are provided:

**For information quality (strongest unique predictor, β = 0.367, p < 0.001):**

a. Invest in high-resolution, multi-angle product images
b. Provide detailed product specifications (dimensions, materials, compatibility)
c. Update inventory information in real time to prevent “out of stock” disappointments
d. Include customer reviews and ratings prominently
e. Use video demonstrations for complex products

**For service quality (largest standardized coefficient in multivariate, β = 0.431):**

a. Implement 24/7 customer support chat (automated for common queries, human for complex issues)
b. Clearly communicate return, refund, and warranty policies before purchase
c. Acknowledge customer inquiries within 2 hours (Iraqi customers expect rapid response)
d. Provide tracking information for all shipments
e. Follow up after delivery to confirm satisfaction

**For system quality (not significant in multivariate, but still important as hygiene factor):**

a. Maintain acceptable levels of system quality (page load speed <3 seconds, uptime >99%)
b. Do NOT over-invest in system quality beyond the “acceptable” threshold
c. Focus resources on information and service quality as differentiators
d. Regularly monitor system quality to ensure it does not fall below the hygiene threshold (which would cause dissatisfaction)

**For marketing strategy (based on H _1_ finding of no gender difference):**

a. Develop gender-neutral marketing campaigns
b. Avoid gender-based segmentation in online advertising
c. Focus on universal appeals (information transparency, service reliability, product quality)

### 5.5 Limitations

1. **Cross-sectional design:** Data were collected at a single time point, preventing causal inferences. Longitudinal research is needed to establish temporal precedence.
2. **Convenience sampling:** The sample may not be fully representative of all Iraqi online shoppers. Customers who are less digitally active or less willing to respond to surveys may have different perceptions.
3. **Self-reported data:** All measures are based on self-report, raising the possibility of common method bias. However, the HTMT results (all <0.85) suggest that common method bias is not severe ( Podsakoff et al., 2003).
4. **Geographic limitation:** The study was conducted only in Baghdad Governorate. Online shopping perceptions may differ in other regions of Iraq.
5. **Single-country context:** Findings may not generalize to other emerging markets with different cultural or infrastructural characteristics.
6. **Aggregated dependent variable in hypothesis testing:** While the conceptual framework includes three dimensions of perceptual attractiveness (EA, WP, CWP), the hypothesis tests used the aggregated score due to sample size limitations for dimension-specific multivariate analysis. Future research with larger samples should test effects on each dimension separately.

## 6. Conclusions and Future research

### 6.1 Conclusions

This study examined the impact of online store specifications (information quality, system quality, service quality) on the attractiveness of customer perception of the product among 350 customers of ten Iraqi online stores. Using a two-stage analytical approach (PLS-SEM for measurement validation, multiple regression with VIF for hypothesis testing), the study reached the following conclusions:

1. **Customer awareness** of online store specifications is moderate (M = 3.592/5), indicating significant room for improvement, particularly in service quality (the lowest-rated dimension).
2. **No gender differences** exist in purchase frequency, suggesting that gender-based segmentation is unnecessary for Iraqi online stores.
3. **Bivariate analysis** confirms that all three dimensions individually have significant positive effects on perceptual attractiveness.
4. **Multivariate analysis** reveals that information quality and service quality retain significance (β = 0.367 and β = 0.431, respectively), while system quality becomes non-significant (β = −0.031, p = 0.493) due to shared variance with the other dimensions.
5. **Herzberg’s Two-Factor Theory** provides a useful framework: information and service quality function as motivator factors (directly enhancing attractiveness), while system quality functions as a hygiene factor (necessary but not sufficient).
6. **Multicollinearity reporting** (VIF and HTMT) is essential in e-commerce quality research to distinguish between true non-significance and statistical suppression due to shared variance.

### 6.2 Recommendations (Linked Directly to Findings)

**Table:** 

Finding	Recommendation	Priority
Information quality has strongest unique effect (β = 0.367)	Invest in high-resolution images, detailed specifications, real-time inventory, customer reviews	High
Service quality has largest standardized coefficient (β = 0.431)	Implement 24/7 chat, clear return policies, rapid response (<2 hours), post-purchase follow-up	High
System quality non-significant in multivariate (β = −0.031)	Maintain acceptable levels (load speed <3 s, uptime >99%) but do NOT over-invest beyond threshold	Medium
Moderate awareness of all dimensions (M = 3.59)	Communicate improvements to customers through marketing channels	Medium
No gender difference in purchase frequency (p = 0.442)	Use gender-neutral marketing strategies	Low

### 6.3 Future research directions

1. **Longitudinal studies:** Track how the effects of IQ, SQ, and SEQ change over time as customers gain experience with online stores and as the Iraqi e-commerce market matures.
2. **Cross-cultural replication:** Replicate this study in other emerging markets (Jordan, Egypt, Saudi Arabia) with similar cultural characteristics (collectivism, high uncertainty avoidance) but different digital infrastructure levels.
3. **Experimental designs:** Use randomized experiments to manipulate information quality (e.g., complete vs. incomplete descriptions) and measure causal effects on perceptual attractiveness.
4. **Dimension-specific dependent variables:** With larger samples (n > 500), test the effects of IQ, SQ, and SEQ separately on each dimension of perceptual attractiveness (EA, WP, CWP) to determine whether different store specifications affect different perceptual components.
5. **Moderator analysis:** Examine whether the effects of store specifications are moderated by customer characteristics (age, education, prior e-commerce experience) or product characteristics (search goods vs. experience goods, price level).
6. **Qualitative research:** Conduct interviews or focus groups with Iraqi online shoppers to understand why they prioritize information and service quality over system quality.
7. **Technology acceptance:** Integrate TAM variables (perceived ease of use, perceived usefulness) with the three-dimensional quality framework to examine mediated pathways.
8. **Comparative store analysis:** Compare the specification-perception relationship across the ten stores individually to identify best practices and underperformers.

## Ethical considerations

This study involved human participants and was conducted in accordance with accepted ethical research standards and the principles outlined in the Declaration of Helsinki. Ethical approval was obtained from the Scientific Research Ethics Committee, University of Fallujah, Iraq (Approval No. HOF.HUM.2025.001). Written informed consent was obtained from all participants prior to their participation. All participants were informed about the purpose of the study, the voluntary nature of their participation, their right to withdraw at any time without consequences, and the confidentiality of their data.

## Data availability

The data supporting the findings of this study are openly available in Zenodo at: **https://doi.org/10.5281/zenodo.20288003** Awni, S., Hammadi, A., Al-halboosi, I., Shakhatreh, H., Salman, D., ababneh,. ayat., Stavytskyy, A., azzam,. farouq., & Shakaterh, R. (2026). The impact of online store specifications on enhancing the attractiveness of customer perception of the product: An analytical study of the opinions of a sample of Iraqi virtual store customers.

These data are available under the terms of the Creative Commons Zero “No rights reserved” data waiver (CC0 1.0 Public Domain Dedication).

### Reporting guidelines

This study is an observational survey-based research and follows the STROBE reporting guidelines. No CONSORT or ARRIVE checklists are required, as the study does not involve clinical trials or animal experiments.
