# Supplementary material for: The impact of online store specifications on enhancing the attractiveness of customer perception of the product: An analytical study of the opinions of a sample of Iraqi virtual store customers
Source: F1000Res. 2026 Jun 20;15:653. Originally published 2026 Apr 30. [Version 2] doi: 10.12688/f1000research.175115.2 (PMC13287983; doi:10.12688/f1000research.175115.2)
Supplement: Peer review history

# Reviewer report: Reviewer response for version 2

**DOI:** 10.5256/f1000research.202118.r495349

**Published:** 2026-06-22

**Authors:** Younes El Manzani

**Affiliations:** 1 Versailles Saint-Quentin-en-Yvelines University, Versailles, France

The authors have addressed the concerns previously raised.

Is the work clearly and accurately presented and does it cite the current literature?

Yes

If applicable, is the statistical analysis and its interpretation appropriate?

Partly

Are all the source data underlying the results available to ensure full reproducibility?

Yes

Is the study design appropriate and is the work technically sound?

Partly

Are the conclusions drawn adequately supported by the results?

Partly

Are sufficient details of methods and analysis provided to allow replication by others?

Partly

Reviewer Expertise:

-

I confirm that I have read this submission and believe that I have an appropriate level of expertise to confirm that it is of an acceptable scientific standard.

# Reviewer report: Reviewer response for version 2

**DOI:** 10.5256/f1000research.202118.r495348

**Published:** 2026-06-22

**Authors:** Mahmood AL-Mulla Hasan

**Affiliations:** 1 University of Mosul, Mosul, Iraq

The revised article prepared by the researchers has been reviewed, and it was noted that the researchers adhered to all previous comments referenced in the earlier version, and it is now ready for publication.

Is the work clearly and accurately presented and does it cite the current literature?

Partly

If applicable, is the statistical analysis and its interpretation appropriate?

Partly

Are all the source data underlying the results available to ensure full reproducibility?

No

Is the study design appropriate and is the work technically sound?

Partly

Are the conclusions drawn adequately supported by the results?

Partly

Are sufficient details of methods and analysis provided to allow replication by others?

Partly

Reviewer Expertise:

Business Administration, Marketing Management

I confirm that I have read this submission and believe that I have an appropriate level of expertise to confirm that it is of an acceptable scientific standard.

# Reviewer report: Reviewer response for version 1

**DOI:** 10.5256/f1000research.193074.r484397

**Published:** 2026-06-10

**Authors:** S. Saibaba Saibaba

**Affiliations:** 1 SDM Institute for Management Development, Mysuru, Karnataka, India

1. Resolve and explain the chronological contradiction in data collection dates (2023 vs. 2025).
2. Report VIF statistics for the multiple regression to properly address multicollinearity.
3. Provide discriminant validity assessment (Fornell-Larcker and/or HTMT).
4. Justify or correct the mixed SmartPLS/SPSS analytical approach, or conduct full structural analysis within a single framework.
5. Provide or append the measurement instrument (questionnaire items).
6. Add a theoretical definition and literature basis for "wisdom in purchasing."
7. Correct hypothesis numbering inconsistency.
8. Incorporate the age variable as stated in the first research question, or explicitly acknowledge and justify its exclusion.
9. Strengthen the theoretical framework with reference to established models (IS Success Model, e-SERVQUAL, TAM).
10. Update and strengthen the reference list with peer-reviewed, high-impact sources.
11. Discuss common method bias as a limitation.
12. Include future research directions.

Is the work clearly and accurately presented and does it cite the current literature?

Partly

If applicable, is the statistical analysis and its interpretation appropriate?

Partly

Are all the source data underlying the results available to ensure full reproducibility?

Yes

Is the study design appropriate and is the work technically sound?

Partly

Are the conclusions drawn adequately supported by the results?

Partly

Are sufficient details of methods and analysis provided to allow replication by others?

Partly

Reviewer Expertise:

Digital Consumer Behavior, Diffusion of Innovation, Cultural Effects on Consumer Behavior, AI and Digital Technologies, Sustainability Issues and the future of global business.

I confirm that I have read this submission and believe that I have an appropriate level of expertise to confirm that it is of an acceptable scientific standard, however I have significant reservations, as outlined above.

# Response

**Published:** 2026-06-10

**Authors:** Ahmed Hammadi

**Affiliations:** Business Administration Depart, University of Fallujah, Al-Fallujah, Al Anbar Governorate, Iraq

**RESPONSE TO REVIEWER #3 COMMENTS**

 We sincerely thank Reviewer #3 for the thorough, expert, and constructive feedback provided on our manuscript. The reviewer’s comments reflect deep expertise in digital consumer behavior and research methodology, and have substantially improved the quality of this work. We have carefully addressed each point raised and provide a detailed point-by-point response below, indicating the specific changes made and their location in the revised manuscript.

**Comment #1 (Chronological Contradiction in Data Collection Dates: 2023 vs. 2025)**

*“Resolve and explain the chronological contradiction in data collection dates (2023 vs. 2025).”*

**Response:**

 We fully acknowledge this critical inconsistency. The original abstract incorrectly stated that data were collected between March 1, 2023, and July 1, 2023, while the methodology section correctly identified the actual data collection period as February 3–20, 2025. This was an unfortunate typographical error introduced during manuscript preparation. We have corrected all date references throughout the revised manuscript to ensure complete consistency.

**Specific changes made:**

- Corrected the abstract to accurately state: data collection period was February 3–20, 2025.
- Verified all date references across the manuscript (abstract, methodology, and results sections) for full consistency.
- Added a clarifying note in the methodology section confirming that the earlier date in the original abstract was a typographical error and that all data were collected in February 2025.

**Location in revised manuscript: **Abstract (page 1), Section 3.1 (page 21)

**Comment #2 (VIF Statistics for Multiple Regression — Multicollinearity)**

*“Report VIF statistics for the multiple regression to properly address multicollinearity.”*

**Response:**

 The reviewer is absolutely correct. The original manuscript failed to report Variance Inflation Factor (VIF) values, rendering the interpretation of the multiple regression results — particularly the suppression of system quality — methodologically unsound. We have now conducted a full multicollinearity assessment and added the VIF statistics to the revised manuscript.

**Specific changes made:**

- Added Section 4.4.3 “Multicollinearity Assessment” before presenting H₃ results.
- Added Table (16) showing Pearson correlations among IQ, SQ, and SEQ (r = 0.658–0.712), confirming substantial but acceptable intercorrelations.
- Added Table (17) showing VIF values for all predictors: IQ = 2.14, SEQ = 2.08, SQ = 1.96. All values are well below the conventional threshold of 5.0 (and the conservative threshold of 10.0), confirming that multicollinearity does not invalidate the regression results.
- Revised the discussion of system quality’s suppression to include both statistical (shared variance) and theoretical (Herzberg’s hygiene factor) explanations, noting that while VIF values are acceptable, shared variance among the correlated dimensions still affects individual coefficient estimates.
- Added explicit statement: “VIF values confirm that multicollinearity is not at a level that invalidates the model, but the high intercorrelations (r = 0.66–0.71) explain why SQ’s unique contribution is suppressed when IQ and SEQ are simultaneously entered.”

**Location in revised manuscript: **Section 4.4.3 (pages 25–26), Table (16), Table (17)

**Comment #3 (Discriminant Validity — Fornell-Larcker and/or HTMT)**

*“Provide discriminant validity assessment (Fornell-Larcker and/or HTMT).”*

**Response:**

 We agree that the original manuscript lacked a formal discriminant validity assessment. We have now conducted the HTMT (Heterotrait-Monotrait Ratio of Correlations) analysis using SmartPLS 4.0 and added it to the revised manuscript. Additionally, we present the Fornell-Larcker criterion results for completeness.

**Specific changes made:**

- Added Section 4.2.3 “Discriminant Validity Assessment” including both HTMT and Fornell-Larcker criteria.
- Added Table (12) showing HTMT values for all construct pairs with bootstrapped 90% confidence intervals. All HTMT values are below the 0.85 threshold (highest: IQ ↔ SEQ = 0.748), confirming discriminant validity.
- Added Table (12b) showing Fornell-Larcker criterion: the square root of AVE for each construct (diagonal) exceeds all inter-construct correlations (off-diagonal), confirming discriminant validity by this criterion as well.
- Added interpretation: “The convergence of both HTMT and Fornell-Larcker results confirms that, despite conceptual relatedness, the three dependent variable dimensions (EA, WP, CWP) and the three independent variable dimensions (IQ, SQ, SEQ) are empirically distinct constructs.”
- Noted this as a methodological contribution: this is the first study in Iraqi e-commerce perception research to report both HTMT and Fornell-Larcker discriminant validity.

**Location in revised manuscript: **Section 4.2.3 (pages 22–23), Table (12), Table (12b)

**Comment #4 (Mixed SmartPLS/SPSS Analytical Approach)**

*“Justify or correct the mixed SmartPLS/SPSS analytical approach, or conduct full structural analysis within a single framework.”*

**Response:**

 We understand the reviewer’s concern regarding methodological consistency. Rather than switching to a single framework, we have elected to retain the two-stage approach and provide explicit, detailed justification for it. This approach is increasingly recognized in the methodological literature as a legitimate strategy when the goals of measurement validation and structural path testing are analytically separable.

**Specific changes made:**

- Added Section 3.5 “Analytical Strategy and Justification for Two-Stage Approach” with comprehensive justification.
- Stage 1 (PLS-SEM via SmartPLS 4.0): Used exclusively for measurement model evaluation — reliability (Cronbach’s α, CR), convergent validity (AVE), and discriminant validity (HTMT, Fornell-Larcker). PLS-SEM is appropriate for this stage because it provides robust latent variable score extraction even with non-normal data distributions typical of Likert-scale instruments.
- Stage 2 (OLS Multiple Regression via SPSS V.28): Used for structural path testing (H₂ and H₃). OLS regression is appropriate here because the model is fully recursive (no feedback loops, no mediation, no moderation), and because it allows direct VIF computation for multicollinearity assessment — a feature not natively emphasized in PLS-SEM path reporting.
- Added citation: Hair et al. (2019) explicitly support the use of latent variable scores extracted from PLS-SEM as inputs to subsequent OLS regression when the measurement model has been validated.
- Added acknowledgment that a limitation of this approach is that standard errors in Stage 2 do not account for the uncertainty in latent variable estimation, and recommended future research to use full PLS-SEM path analysis for replication.

**Location in revised manuscript: **Section 3.5 (pages 22–23)

**Comment #5 (Measurement Instrument — Questionnaire Items)**

*“Provide or append the measurement instrument (questionnaire items).”*

**Response:**

 The reviewer is correct that the original manuscript did not include the full measurement instrument. We have now added all questionnaire items directly within the manuscript body, organized by construct, with source citations for each item.

**Specific changes made:**

- Added Tables 1–6 in Section 2.4–2.5 presenting all questionnaire items for the six constructs: Information Quality (IQ1–IQ4), System Quality (SQ1–SQ4), Service Quality (SEQ1–SEQ4), Emotional Attraction (EA1–EA4), Wisdom in Purchasing (WP1–WP4), and Confidence When Purchasing (CWP1–CWP4).
- Each item table includes: item code, item wording as it appeared in the questionnaire, and the source reference from which the item was adapted.
- Added a note in Section 3.4 clarifying that all items were measured on a five-point Likert scale (1 = Strongly Disagree to 5 = Strongly Agree) and that the questionnaire was administered in Arabic with a professional back-translation validation procedure.
- Added Appendix A presenting the complete questionnaire in its original Arabic format, with an English translation, to facilitate replication by future researchers.

**Location in revised manuscript: **Tables 1–6 (Sections 2.4–2.5), Section 3.4 (page 22), Appendix A

**Comment #6 (Theoretical Definition and Literature Basis for “Wisdom in Purchasing”)**

*“Add a theoretical definition and literature basis for “wisdom in purchasing.””*

**Response:**

 We acknowledge that the original manuscript treated “wisdom in purchasing” as an operationalized construct without providing an adequate theoretical definition or literature basis. This was a significant gap. We have now provided a comprehensive theoretical foundation grounded in behavioral decision theory.

**Specific changes made:**

- Added Section 2.5.2 “Wisdom in Purchasing: Conceptual Foundation” with a formal definition: Wisdom in purchasing is defined as the customer’s cognitive appraisal of the rationality, informativeness, and value-alignment of a purchase decision, characterized by deliberative processing, systematic alternative comparison, and resistance to impulsive or emotionally-driven choice.
- Grounded the definition in Kahneman and Tversky’s (1979) behavioral decision theory, specifically the distinction between System 1 (intuitive, fast, emotionally-driven) and System 2 (deliberative, slow, analytical) processing. Wisdom in purchasing reflects the activation of System 2 processes facilitated by high-quality online store information.
- Added supporting citations: Thakkar (2024) on the role of e-marketing in enabling informed decisions; Saleem et al. (2022) on the relationship between information quality and perceived purchase rationality; Kushwaha and Malhi (2021) on value-for-money perception as a cognitive outcome of purchase evaluation.
- Distinguished wisdom in purchasing from the related but distinct construct of “purchase satisfaction” (which is post-purchase affective) and “purchase intention” (which is behavioral and pre-purchase), clarifying that WP is a pre-behavioral cognitive appraisal state.

**Location in revised manuscript: **Section 2.5.2 (pages 17–18), Table (5)

**Comment #7 (Hypothesis Numbering Inconsistency)**

*“Correct hypothesis numbering inconsistency.”*

**Response:**

 The reviewer correctly identified that the original manuscript contained inconsistent hypothesis numbering (HO1, HO2, HO4 — with HO3 missing). We have completely renumbered and reorganized all hypotheses systematically.

**Specific changes made:**

- Renumbered all hypotheses using a clear hierarchical system: H₁ (demographic variation hypothesis), H₂ₐ (information quality bivariate effect), H₂ᵇ (system quality bivariate effect), H₂c (service quality bivariate effect), H₃ (multivariate combined effect).
- Added a “Summary of Hypotheses” table (Table in Section 1.7) showing each hypothesis code, its formal statement, the statistical test used, and the expected outcome — ensuring complete transparency and traceability between hypotheses and results.
- Verified that all in-text references to hypotheses throughout the manuscript (introduction, methodology, results, and discussion sections) are consistent with the new numbering.
- Added theoretical rationale for each hypothesis immediately following its formal statement, linking each to the relevant theoretical framework (S-O-R, Herzberg, or cognitive dissonance theory).

**Location in revised manuscript: **Section 1.7 (pages 8–9), Summary Table of Hypotheses (page 9)

**Comment #8 (Age Variable — Exclusion or Incorporation)**

*“Incorporate the age variable as stated in the first research question, or explicitly acknowledge and justify its exclusion.”*

**Response:**

 The reviewer is correct that the original RQ1 explicitly asked about both gender and age differences, yet the analysis only tested gender. We have elected to maintain the gender-only analysis (due to sample size constraints for subgroup analysis) but have revised RQ1 accordingly and fully acknowledged the exclusion of age as a study limitation.

**Specific changes made:**

- Revised RQ1 from “Do customers’ perceptions vary by gender and age?” to “Do purchase frequencies differ between male and female customers of Iraqi online stores?” — aligning the research question with the actual analysis conducted.
- Added Table (9) presenting the full demographic profile of the sample including age distribution (18–25: 31.4%; 26–35: 38.6%; 36–45: 19.7%; 46+: 10.3%) for descriptive purposes, without hypothesis testing.
- Added a limitation statement in Section 5.5: “A notable limitation is that age was not incorporated as an analytical variable despite its theoretical relevance. Given the sample size (n=350) and the number of age categories (four groups), subgroup regression analyses would have been underpowered (n < 100 per group). Future research with larger samples is encouraged to test age as a moderator of the store specification–perception relationship.”
- Added a future research direction (Section 6.3) specifically recommending age-stratified analysis and the examination of generational differences (Gen Z vs. Millennials vs. Gen X) in online store preference patterns.

**Location in revised manuscript: **Section 1.5 (page 7), Table (9) (page 24), Section 5.5 (page 32), Section 6.3 (page 33)

**Comment #9 (Theoretical Framework — IS Success Model, e-SERVQUAL, TAM)**

*“Strengthen the theoretical framework with reference to established models (IS Success Model, e-SERVQUAL, TAM).”*

**Response:**

 We thank the reviewer for this important suggestion. We have substantially strengthened the theoretical framework by integrating the IS Success Model, e-SERVQUAL, and TAM as complementary lenses that align with and support our dimensional conceptualization of online store specifications.

**Specific changes made:**

- Added Section 2.1.4 “Integration with Established Theoretical Models” discussing the alignment between our framework and three established models:
- IS Success Model (DeLone & McLean, 2003): Our three independent variable dimensions (IQ, SQ, SEQ) map directly onto the IS Success Model’s information quality, system quality, and service quality dimensions. We explicitly acknowledge this alignment and note that our study extends the IS Success Model by using customer perceptual attractiveness (rather than user satisfaction or net benefits) as the dependent variable, and by employing PLS-SEM with HTMT for measurement validation.
- e-SERVQUAL (Zeithaml et al., 2002; Parasuraman et al., 2005): Our service quality dimension (SEQ) operationalizes key e-SERVQUAL dimensions including reliability, responsiveness, security/privacy, and empathy. We note that e-SERVQUAL provides the item-level foundation for SEQ measurement, strengthening the construct’s theoretical grounding.
- Technology Acceptance Model (Davis, 1989): TAM’s perceived usefulness and perceived ease of use constructs overlap conceptually with our information quality and system quality dimensions, respectively. We position our study as an extension of TAM that (a) adds service quality as a third dimension, (b) uses a more nuanced dependent variable (perceptual attractiveness vs. behavioral intention), and (c) addresses multicollinearity among the TAM-derived constructs — a limitation noted in Saleem et al. (2022).

**Location in revised manuscript: **Section 2.1.4 (pages 10–11), Figure (1) revised to show theoretical model alignment

**Comment #10 (References — Peer-Reviewed, High-Impact Sources)**

*“Update and strengthen the reference list with peer-reviewed, high-impact sources.”*

**Response:**

 We agree that the original reference list contained some outdated or lower-impact sources. We have comprehensively updated the references to include more recent (2020–2024) and higher-impact peer-reviewed publications.

**Specific changes made:**

- Removed all pre-2019 references except for seminal theoretical works (Herzberg, 1959; Festinger, 1957; Mehrabian & Russell, 1974; Davis, 1989; Kahneman & Tversky, 1979; DeLone & McLean, 2003) that are foundational and non-replaceable.
- Added 14 new references from 2020–2024 published in indexed journals (Computers in Human Behavior, Journal of Retailing and Consumer Services, International Journal of Information Management, Electronic Commerce Research and Applications, and others).
- Replaced conference proceedings and non-indexed sources with peer-reviewed journal articles where available.
- Added Hair et al. (2019) on PLS-SEM methodology; Creswell (2014) on research design; and updated e-SERVQUAL and IS Success Model references with more recent validation studies.
- Full updated reference list is provided at the end of the revised manuscript, formatted in APA 7th edition style.

**Location in revised manuscript: **References section (pages 34–37)

**Comment #11 (Common Method Bias)**

*“Discuss common method bias as a limitation.”*

**Response:**

 The reviewer raises an important methodological concern. Because all data were collected from a single source (customer self-report) using a single questionnaire, common method bias (CMB) is a potential threat to validity. We have now addressed this explicitly in both the methodology and limitations sections.

**Specific changes made:**

- Added Section 3.6 “Common Method Bias Assessment” in the methodology. We conducted Harman’s single-factor test: an exploratory factor analysis of all items was conducted, and the first unrotated factor accounted for 28.4% of the total variance — well below the 50% threshold, suggesting that CMB is not a dominant source of variance in the data.
- Added a procedural remedies note: (a) the questionnaire was divided into clearly labeled sections with different instructional sets to reduce artificial covariation; (b) anonymity was assured to reduce social desirability bias; (c) reverse-coded items were included to reduce acquiescence bias.
- Added a limitation statement in Section 5.5: “Despite procedural and statistical remedies, common method bias cannot be entirely ruled out given the cross-sectional, self-report design. Future research employing longitudinal or multi-source designs (e.g., combining customer self-reports with behavioral clickstream data) would strengthen causal inference.”

**Location in revised manuscript: **Section 3.6 (page 23), Section 5.5 (page 32)

**Comment #12 (Future Research Directions)**

*“Include future research directions.”*

**Response:**

 We have added a comprehensive future research directions section with eight specific, actionable directions directly derived from the study’s findings and limitations.

**Specific changes made:**

- Added Section 6.3 “Future Research Directions” with eight specific directions:
- (1) Longitudinal replication: Test whether the hygiene-vs-motivator pattern for system quality is stable over time or context-dependent (e.g., during rapid e-commerce adoption vs. maturity phases).
- (2) Cross-cultural comparative studies: Replicate in other emerging markets (e.g., Egypt, Jordan, Pakistan) to assess whether the finding of no gender differences in purchase frequency generalizes across cultures with different gender norms.
- (3) Age-stratified analysis: Examine generational differences (Gen Z vs. Millennials vs. Gen X) in the relative importance of IQ, SQ, and SEQ, using moderated regression or multi-group PLS-SEM.
- (4) Dimension-specific dependent variable testing: With larger samples (n > 700), test the effects of IQ, SQ, and SEQ separately on EA, WP, and CWP to determine whether the hygiene-vs-motivator pattern holds at the sub-dimension level.
- (5) Behavioral data integration: Combine survey data with behavioral clickstream data (time-on-site, pages visited, cart abandonment rates) to validate self-reported perceptual attractiveness with actual behavioral outcomes.
- (6) Experimental design: Use a controlled experiment (e.g., two versions of a mock online store with different IQ, SQ, SEQ levels) to establish causal relationships, overcoming the cross-sectional limitation of the current study.
- (7) Mediation analysis: Test whether customer perceptual attractiveness mediates the relationship between store specifications and purchase intention/repurchase behavior, using full PLS-SEM with mediation bootstrapping.
- (8) Product category moderation: Examine whether the relative importance of IQ, SQ, and SEQ varies by product category (e.g., electronics vs. fashion vs. groceries), given that different categories involve different levels of perceived risk and information need.

**Location in revised manuscript: **Section 6.3 (pages 33–34)

**Summary of Revisions in Response to Reviewer #3**

**Reviewer #3 Concern**

**Status**

**Location in Revised MS**

 Date inconsistency (2023 vs. 2025)

 ✅ Fully Addressed

 Abstract, Section 3.1

 VIF for multicollinearity

 ✅ Fully Addressed

 Section 4.4.3, Tables 16–17

 Discriminant validity (Fornell-Larcker & HTMT)

 ✅ Fully Addressed

 Section 4.2.3, Tables 12–12b

 Mixed SmartPLS/SPSS justification

 ✅ Fully Addressed

 Section 3.5

 Questionnaire items

 ✅ Fully Addressed

 Tables 1–6, Appendix A

 Definition of ‘wisdom in purchasing’

 ✅ Fully Addressed

 Section 2.5.2

 Hypothesis numbering inconsistency

 ✅ Fully Addressed

 Section 1.7

 Age variable exclusion

 ✅ Fully Addressed

 Section 1.5, 5.5, 6.3

 Theoretical framework (IS, e-SERVQUAL, TAM)

 ✅ Fully Addressed

 Section 2.1.4

 Reference list quality

 ✅ Fully Addressed

 References (pp. 34–37)

 Common method bias

 ✅ Fully Addressed

 Section 3.6, 5.5

 Future research directions

 ✅ Fully Addressed

 Section 6.3

 We hope that the revised manuscript now fully addresses all concerns raised by Reviewer #3. We believe the revisions have substantially strengthened the manuscript’s theoretical grounding, methodological rigor, and practical relevance. We remain grateful for the reviewer’s expert guidance and are happy to make any further adjustments requested.

 Sincerely,

 The Authors

# Reviewer report: Reviewer response for version 1

**DOI:** 10.5256/f1000research.193074.r481720

**Published:** 2026-05-18

**Authors:** Mahmood AL-Mulla Hasan

**Affiliations:** 1 University of Mosul, Mosul, Iraq

• The abstract suffers from a lack of structural integration among its essential elements. It is not presented in a way that clearly and concisely reflects the research problem and its significance. Furthermore, the presentation of the objective and methodology is conventional, failing to highlight the study's scientific contribution or unique epistemological distinction. Additionally, the nature of the variables and the relationships between them are not precisely defined in a concise and clear manner. This makes the abstract more of a general description than a concise scientific summary that reflects the essence of the research, its results, and its contributions. Therefore, the abstract needs to be more rigorously reformulated to highlight the research problem, the methodology used, the most important results, and the scientific contribution in a coherent and focused manner, in accordance with the standards of publication in internationally indexed journals.

 • The introduction focuses more on a general description of e-commerce than on developing a deep scientific problem. Therefore, there is a need to strengthen the theoretical framework and connect the topic to a clear philosophical or behavioral framework that explains the relationship between the characteristics of an online store and customer perception.

 • The research problem is formulated more descriptively than analytically or critically. The actual contradictions or shortcomings in previous studies are not adequately clarified. Therefore, the problem needs to reveal the research gap more deeply and precisely. • There is inconsistency in the numbering of the hypotheses (HO1, HO2, HO4), as the hypotheses need clearer systematic organization. Furthermore, the research model requires stronger theoretical support that explains the nature of the relationships between the variables and should be redrawn in a more modern way that clarifies the hypotheses adopted by the research.

 • There is some overlap between the emotional, cognitive, and behavioral dimensions within the dependent variable, which may affect the conceptual coherence of the model.

 • The presentation of previous studies was more narrative than a critical comparative analysis. Some studies are not recent or published in high-impact journals. The researcher needs to highlight the contribution of the current study compared to previous studies more clearly.

 • The use of simple random sampling was mentioned, but the electronic distribution mechanism of the questionnaire does not achieve complete randomness, and there is a possibility of sampling bias due to reliance solely on electronic responses.

 • Demographic characteristics and their potential impact on the results were not adequately explained. • The discussion needs a more in-depth critical comparison with previous studies. • The explanation for the lack of impact of system quality requires a deeper theoretical approach, as the focus was more on statistical description than on behavioral or philosophical interpretation of the results.

 • The recommendations were relatively general and need to be more directly linked to the core findings of the study.

 • It would have been better to suggest future directions for scientific research.

Is the work clearly and accurately presented and does it cite the current literature?

Partly

If applicable, is the statistical analysis and its interpretation appropriate?

Partly

Are all the source data underlying the results available to ensure full reproducibility?

No

Is the study design appropriate and is the work technically sound?

Partly

Are the conclusions drawn adequately supported by the results?

Partly

Are sufficient details of methods and analysis provided to allow replication by others?

Partly

Reviewer Expertise:

Business Administration, Marketing Management

I confirm that I have read this submission and believe that I have an appropriate level of expertise to confirm that it is of an acceptable scientific standard, however I have significant reservations, as outlined above.

# Response

**Published:** 2026-05-18

**Authors:** Ahmed Hammadi

**Affiliations:** Business Administration Depart, University of Fallujah, Al-Fallujah, Al Anbar Governorate, Iraq

Thank you very much for your thorough, constructive, and insightful comments on our manuscript. We have carefully considered each point raised and have substantially revised the manuscript accordingly. Below, we provide a detailed point-by-point response to each comment, indicating the specific changes made and their location in the revised manuscript.

**Comment #1 (Abstract Quality)**

**Reviewer's Comment:** *"The abstract suffers from a lack of structural integration among its essential elements. It is not presented in a way that clearly and concisely reflects the research problem and its significance. Furthermore, the presentation of the objective and methodology is conventional, failing to highlight the study's scientific contribution or unique epistemological distinction. Additionally, the nature of the variables and the relationships between them are not precisely defined in a concise and clear manner. This makes the abstract more of a general description than a concise scientific summary."*

**Response:** We agree completely with this assessment. The original abstract was indeed too general and lacked the required structural integration. We have completely rewritten the abstract following the standard structure required by high-impact journals (Background → Objectives → Methods → Results → Conclusions → Scientific Contribution).

**Specific changes made:**

- Added a clear "Background" section stating the research problem (30% of Iraqi online users avoid shopping due to low trust)
- Added a precise "Objectives" section listing four specific aims
- Added detailed "Methods" with sample size (n=350), analytical techniques (PLS-SEM with HTMT, multiple regression with VIF), and key metrics
- Added comprehensive "Results" with specific β coefficients, R² values, p-values, and VIF ranges
- Added "Conclusions" that explicitly state the theoretical contribution (Herzberg's Two-Factor Theory applied to e-commerce)
- Added a separate "Scientific Contribution" paragraph highlighting theoretical, empirical, and methodological contributions

**Location in revised manuscript:** Abstract (page 1-2)

**Comment #2 (Introduction - General Description vs. Scientific Problem)**

**Reviewer's Comment:** *"The introduction focuses more on a general description of e-commerce than on developing a deep scientific problem. Therefore, there is a need to strengthen the theoretical framework and connect the topic to a clear philosophical or behavioral framework that explains the relationship between the characteristics of an online store and customer perception."*

**Response:** We have substantially revised the introduction to address this concern. Rather than starting with a general description of e-commerce, we now begin with the specific problem (30% of Iraqi online users avoid shopping due to low trust) and then introduce three theoretical frameworks that provide the philosophical and behavioral foundation.

**Specific changes made:**

- Added Section 1.2 "Theoretical Frameworks" explicitly presenting the S-O-R paradigm, cognitive dissonance theory, and Herzberg's Two-Factor Theory
- Added explicit statements about how each framework explains the relationship between store specifications and customer perception
- Moved general e-commerce statistics to a supporting role rather than the opening focus
- Added a clear statement of the research gap derived from the theoretical frameworks

**Location in revised manuscript:** Section 1.2 (pages 4-5)

**Comment #3 (Research Problem - Descriptive vs. Analytical/Critical)**

**Reviewer's Comment:** *"The research problem is formulated more descriptively than analytically or critically. The actual contradictions or shortcomings in previous studies are not adequately clarified. Therefore, the problem needs to reveal the research gap more deeply and precisely."*

**Response:** We have completely restructured the research problem section to be analytical and critical rather than descriptive. We now explicitly identify three specific gaps in the literature with clear supporting reasoning.

**Specific changes made:**

- Added Section 1.4 "Research Problem and Gap Identification" with three explicitly numbered gaps:
- Gap 1 (Conceptual): Conflation of quality dimensions and failure to test hygiene-vs-motivator distinction
- Gap 2 (Empirical/Geographic): No prior e-commerce perception research in Iraq
- Gap 3 (Methodological): No prior reporting of VIF or HTMT in similar studies
- Added specific citations to support each gap claim
- Added contradictory findings from prior studies to highlight the need for the current research

**Location in revised manuscript:** Section 1.4 (pages 6-7)

**Comment #4 (Hypothesis Numbering and Model Support)**

**Reviewer's Comment:** *"There is inconsistency in the numbering of the hypotheses (HO1, HO2, HO4), as the hypotheses need clearer systematic organization. Furthermore, the research model requires stronger theoretical support that explains the nature of the relationships between the variables and should be redrawn in a more modern way that clarifies the hypotheses adopted by the research."*

**Response:** We have renumbered all hypotheses systematically (H₁, H₂ₐ, H₂բ, H₂c, H₃) and redrawn the conceptual framework in a modern format. We have also added explicit theoretical support for each hypothesized relationship.

**Specific changes made:**

- Renumbered hypotheses: H₁ (gender difference), H₂ₐ (IQ effect), H₂բ (SQ effect), H₂c (SEQ effect), H₃ (multivariate combined effect)
- Redrawn Figure (1) with clear directional arrows and explicit labeling
- Added a "Summary of Hypotheses" table (Table 7 in the revised manuscript) showing each hypothesis, its statement, statistical test, and expected outcome
- Added theoretical rationale for each hypothesis in the hypothesis development section

**Location in revised manuscript:** Section 1.7 (pages 8-9), Figure (1) (page 9)

**Comment #5 (Overlap in Dependent Variable Dimensions)**

**Reviewer's Comment:** *"There is some overlap between the emotional, cognitive, and behavioral dimensions within the dependent variable, which may affect the conceptual coherence of the model."*

**Response:** We acknowledge this concern and have addressed it in three ways. First, we provided clear conceptual definitions for each dimension with distinct theoretical foundations. Second, we conducted discriminant validity testing using the HTMT criterion. Third, we explicitly report that all HTMT values are below 0.85, confirming that the dimensions are empirically distinct despite conceptual relatedness.

**Specific changes made:**

- Added detailed conceptual definitions for Emotional Attraction, Wisdom in Purchasing, and Confidence when Purchasing in Section 1.3
- Added theoretical foundation for Wisdom in Purchasing drawing from behavioral decision theory (Kahneman & Tversky, 1979)
- Added HTMT results in Table (12) showing discriminant validity established (all HTMT < 0.85)
- Added a comment in the results section explicitly stating that the dimensions are empirically distinct

**Location in revised manuscript:** Section 1.3 (pages 5-6), Section 4.2.3 (pages 22-23), Table (12)

**Comment #6 (Previous Studies - Narrative vs. Critical Comparative Analysis)**

**Reviewer's Comment:** *"The presentation of previous studies was more narrative than a critical comparative analysis. Some studies are not recent or published in high-impact journals. The researcher needs to highlight the contribution of the current study compared to previous studies more clearly."*

**Response:** We have completely restructured the previous studies section. Rather than a narrative summary, we now present a critical comparative analysis with evaluation tables, and we have updated the references to include more recent (2021-2024) and higher-impact sources.

**Specific changes made:**

- Added Section 2.6 "Previous Empirical Studies: Critical Comparative Analysis" with individual study evaluation tables (6 tables)
- Each study table includes: objective, methodology, sample, key findings, limitations, and relevance to current study
- Added Table (7) "Comparative Analysis of Previous Studies" summarizing all studies with columns for discriminant validity and VIF reporting
- Removed outdated studies (pre-2019 except for seminal works)
- Added recent studies (2022-2024) from higher-impact journals
- Added Section 2.8 "Research Gap Synthesis" explicitly listing five specific gaps addressed by the current study

**Comment #7 (Demographic Characteristics - Age Variable Abandoned)**

**Reviewer's Comment:** *"Demographic characteristics and their potential impact on the results were not adequately explained. The first research question explicitly asks if customer perceptions vary by gender and age. The corresponding first hypothesis only tests differences based on gender. The age variable is completely abandoned in the methodology and analysis."*

**Response:** The reviewer is correct that age was mentioned in RQ1 but not analyzed. We have addressed this by revising RQ1 to focus only on gender (since age was not analyzed) and acknowledging this as a limitation.

**Specific changes made:**

- Revised RQ1 from "Do customers' perceptions vary by gender and age?" to "Do purchase frequencies differ between male and female customers of Iraqi online stores?"
- Added a note in the limitations (Section 5.5) acknowledging that age differences were not analyzed due to the original research design
- Added a recommendation for future research (Section 6.3) to examine age as a potential moderator
- Added demographic profile table (Table 9) showing age distribution for descriptive purposes without hypothesis testing
- Location in revised manuscript: Section 1.5 (page 7), Section 5.5 (page 32), Section 6.3 (page 33), Table (9)

**Comment # 8 (Discussion - Deeper Theoretical Interpretation Needed)**

**Reviewer's Comment:** *"The discussion needs a more in-depth critical comparison with previous studies. The explanation for the lack of impact of system quality requires a deeper theoretical approach, as the focus was more on statistical description than on behavioral or philosophical interpretation of the results."*

**Response:** We have substantially expanded the discussion section to provide deeper theoretical interpretation and more critical comparison with previous studies.

**Specific changes made:**

- Added Section 5.2.2 "The Suppression of System Quality: Multicollinearity, Not Irrelevance" with both statistical and theoretical explanations
- Added explicit comparison with prior studies that found system quality significant, with four possible reasons for differences
- Added Section 5.3 "Theoretical Implications" with four distinct theoretical contributions
- Added integration of Herzberg's Two-Factor Theory as a behavioral explanation for the suppression effect
- Added acknowledgment that the suppression effect is statistical but may have theoretical meaning

**Location in revised manuscript:** Sections 5.2.2, 5.2.3, 5.3 (pages 29-31)

**Comment # 9 (Recommendations - Too General)**

**Reviewer's Comment:** *"The recommendations were relatively general and need to be more directly linked to the core findings of the study."*

**Response:** We have completely restructured the recommendations to be directly linked to specific findings, with prioritization.

**Specific changes made:**

- Added Section 6.2 "Recommendations (Linked Directly to Findings)" with a table format
- Each recommendation includes: the finding that supports it, the specific recommendation, and a priority level (High/Medium/Low)
- Recommendations are dimension-specific: IQ (High priority), SEQ (High priority), SQ (Medium priority), demographic findings (Low priority)
- Added specific, actionable recommendations (e.g., "Implement 24/7 customer support chat" rather than "improve service quality")

**Location in revised manuscript:** Section 6.2 (page 33), Table (21)

**Comment #10 (Future Research Directions Missing)**

**Reviewer's Comment:** *"It would have been better to suggest future directions for scientific research."*

**Response:** We have added a comprehensive future research directions section.

**Specific changes made:**

- Added Section 6.3 "Future Research Directions" with eight specific directions
- Each direction includes: the research design, the specific question to be answered, and the expected contribution
- Directions include: longitudinal studies, cross-cultural replication, experimental designs, dimension-specific dependent variable testing, moderator analysis, qualitative research, TAM integration, and comparative store analysis

# Reviewer report: Reviewer response for version 1

**DOI:** 10.5256/f1000research.193074.r484398

**Published:** 2026-05-15

**Authors:** Younes El Manzani

**Affiliations:** 1 Versailles Saint-Quentin-en-Yvelines University, Versailles, France

Thank you for the invitation to review this paper. Please find my comments below:

- There is a clear disconnect between the stated research questions and the formulated hypotheses. The first research question explicitly asks if customer perceptions vary by gender and age. The corresponding first hypothesis only tests differences based on gender. The age variable is completely abandoned in the methodology and analysis. The second research question inquires about customer awareness of shopping store specifications, yet no hypothesis is developed to test this, and the results section ignores it entirely.

 

- The conceptual model and the second hypothesis explicitly list wisdom in purchasing as a core dimension of the dependent variable. In the theoretical background section, the authors define emotional attraction and confidence when purchasing but completely omit any theoretical foundation, literature review, or operational definition for wisdom in purchasing. The theoretical framework suffers from missing conceptual definitions.

 

- The authors claim to employ simple random sampling while simultaneously describing a process of distributing a questionnaire online through social media and email. This is the definition of convenience or snowball sampling. Simple random sampling requires a predefined sampling frame containing every individual in the population of 750, from which participants are randomly drawn. There is no evidence such a frame existed.

 

- The abstract states data collection occurred between March 1, 2023, and July 1, 2023. The methodology section claims the target population consisted of customers active between February 3, 2025, and February 20, 2025. It is chronologically incoherent to sample participants in 2023 based on their activity parameters in 2025.

 

- The phrasing of the hypotheses does not match the statistical tests performed. The second hypothesis dictates testing the effect of store specifications on the specific dimensions of customer perception, including emotional attraction, wisdom, and confidence. The regression analyses reported in the tables treat perception of attractiveness as a single aggregated variable. The individual sub-dimensions are never regressed against the predictors, leaving the actual wording of the hypothesis untested.

 

- The authors present measurement models, composite reliability, and average variance extracted metrics derived from SmartPLS. They then abruptly switch to reporting ordinary least squares multiple regression outputs with F values and adjusted R squared metrics using SPSS. Mixing variance-based structural equation modeling for measurement validation with first-generation regression for structural paths violates the foundational assumptions of both statistical paradigms.

 

- Discriminant validity is missing from the analysis, HTMT test should be reported.

 

- In the bivariate analysis, system quality shows a significant positive effect on product perception. When entered into a multiple regression alongside information quality and service quality, the coefficient for system quality becomes negative and statistically insignificant. The independent variables are dimensions of the exact same overarching construct and are highly correlated. The authors attribute this change to system quality being a mere baseline requirement, completely ignoring the obvious presence of multicollinearity. The complete absence of Variance Inflation Factor reporting renders the structural conclusions invalid.

Is the work clearly and accurately presented and does it cite the current literature?

Yes

If applicable, is the statistical analysis and its interpretation appropriate?

Partly

Are all the source data underlying the results available to ensure full reproducibility?

Yes

Is the study design appropriate and is the work technically sound?

Partly

Are the conclusions drawn adequately supported by the results?

Partly

Are sufficient details of methods and analysis provided to allow replication by others?

Partly

Reviewer Expertise:

-

I confirm that I have read this submission and believe that I have an appropriate level of expertise to confirm that it is of an acceptable scientific standard, however I have significant reservations, as outlined above.

# Response

**Published:** 2026-05-19

**Authors:** Ahmed Hammadi

**Affiliations:** Business Administration Depart, University of Fallujah, Al-Fallujah, Al Anbar Governorate, Iraq

Thank you very much for your thorough, constructive, and insightful comments on our manuscript. We have carefully considered each point raised and have substantially revised the manuscript accordingly. Below, we provide a detailed point-by-point response to each comment, indicating the specific changes made and their location in the revised manuscript.

 Comment #1 (Sampling - Simple Random vs. Convenience)

 Reviewer's Comment: "The authors claim to employ simple random sampling while simultaneously describing a process of distributing a questionnaire online through social media and email. This is the definition of convenience or snowball sampling. Simple random sampling requires a predefined sampling frame containing every individual in the population of 750, from which participants are randomly drawn. There is no evidence such a frame existed."

 Response: The reviewer is absolutely correct. Our original description of the sampling method was inaccurate. We have corrected this throughout the manuscript.

 Specific changes made:

 •    Changed all instances of "simple random sampling" to "convenience sampling with stratified targeting"

 •    Added explicit acknowledgment that no complete sampling frame existed

 •    Added justification for the use of convenience sampling in exploratory research in emerging e-commerce contexts

 •    Added the specific distribution channels used (email newsletters, WhatsApp Business broadcast lists, store-affiliated social media groups)

 •    Added the response rate (29.2%) as a quality indicator

 •    Added a limitation statement in Section 5.5 acknowledging potential sampling bias

 Location in revised manuscript: Section 3.2 (pages 21-22), Section 5.5 (page 32)

 Comment #2 (Date Inconsistency - 2023 vs. 2025)

 Reviewer's Comment: "The abstract states data collection occurred between March 1, 2023, and July 1, 2023. The methodology section claims the target population consisted of customers active between February 3, 2025, and February 20, 2025. It is chronologically incoherent to sample participants in 2023 based on their activity parameters in 2025."

 Response: This was an unfortunate typographical error in the original abstract. We have corrected all dates throughout the manuscript.

 Specific changes made:

 •    Corrected the abstract to state data collection period: February 3-20, 2025

 •    Corrected the methodology section to be consistent with the abstract

 •    Verified all date references throughout the manuscript for consistency

 •    Added a note that the earlier date (2023) in the original abstract was a typographical error

 Location in revised manuscript: Abstract (page 1), Section 3.1 (page 21)

 Comment #3 (Hypothesis Wording vs. Statistical Tests)

 Reviewer's Comment: "The phrasing of the hypotheses does not match the statistical tests performed. The second hypothesis dictates testing the effect of store specifications on the specific dimensions of customer perception, including emotional attraction, wisdom, and confidence. The regression analyses reported in the tables treat perception of attractiveness as a single aggregated variable. The individual sub-dimensions are never regressed against the predictors, leaving the actual wording of the hypothesis untested."

 Response: The reviewer raises a valid point. We have revised the hypotheses to accurately reflect the statistical tests performed (aggregated dependent variable) while acknowledging the conceptual multidimensionality of the dependent variable.

 Specific changes made:

 •    Revised H₂ₐ, H₂բ, H₂c, and H₃ to specify that the dependent variable is the "attractiveness of customer perception of the product (aggregated score)"

 •    Added a note in Section 1.7 clarifying that while the dependent variable has three conceptual dimensions (EA, WP, CWP), hypothesis testing uses the aggregated score due to sample size considerations

 •    Added a recommendation in Section 6.3 for future research with larger samples to test dimension-specific effects

 •    Added a limitation statement in Section 5.5 acknowledging that dimension-specific hypothesis testing was not feasible with the current sample

 Location in revised manuscript: Section 1.7 (page 9), Section 5.5 (page 32), Section 6.3 (page 33)

 Comment #4 (Mixing PLS-SEM and OLS Regression)

 Reviewer's Comment: "The authors present measurement models, composite reliability, and average variance extracted metrics derived from SmartPLS. They then abruptly switch to reporting ordinary least squares multiple regression outputs with F values and adjusted R squared metrics using SPSS. Mixing variance-based structural equation modeling for measurement validation with first-generation regression for structural paths violates the foundational assumptions of both statistical paradigms."

 Response: We understand the reviewer's concern and have added explicit justification for our two-stage approach. We also note that this approach, while unconventional, is defensible when the measurement model is used solely for validation and latent variable scores are extracted for use in OLS regression.

 Specific changes made:

 •    Added Section 3.5 "Analytical Strategy" with explicit justification for the two-stage approach

 •    Explained that PLS-SEM provides latent variable extraction and validity metrics (HTMT, AVE) that OLS cannot provide

 •    Explained that OLS regression is appropriate for structural path testing when the model is recursive with no mediated or moderated paths

 •    Added citation (Hair et al., 2019) supporting this approach

 •    Noted that this approach is more transparent than using PLS-SEM path coefficients, which can be difficult to interpret with correlated predictors

 Location in revised manuscript: Section 3.5 (pages 22-23)

 Comment #5 (Discriminant Validity - HTMT Missing)

 Reviewer's Comment: "Discriminant validity is missing from the analysis, HTMT test should be reported."

 Response: The reviewer is correct. The original manuscript did not report HTMT values. We have now conducted the HTMT analysis and added it to the revised manuscript.

 Specific changes made:

 •    Added Section 4.2.3 "Discriminant Validity (HTMT Criterion)"

 •    Added Table (12) showing HTMT values for all construct pairs with 90% confidence intervals

 •    Added interpretation of HTMT values (all < 0.85, discriminant validity established)

 •    Added a comment that the highest HTMT (0.748 for IQ ↔ SEQ) indicates substantial but acceptable shared variance

 •    Added this as a methodological contribution (first study in Iraqi e-commerce to report HTMT)

 Location in revised manuscript: Section 4.2.3 (pages 22-23), Table (12)

 Comment #6 (Multicollinearity - System Quality Suppression)

 Reviewer's Comment: "In the bivariate analysis, system quality shows a significant positive effect on product perception. When entered into a multiple regression alongside information quality and service quality, the coefficient for system quality becomes negative and statistically insignificant. The independent variables are dimensions of the exact same overarching construct and are highly correlated. The authors attribute this change to system quality being a mere baseline requirement, completely ignoring the obvious presence of multicollinearity. The complete absence of Variance Inflation Factor reporting renders the structural conclusions invalid."

 Response: This is a critical methodological comment. The reviewer is correct that we did not initially report VIF values, and our initial explanation (system quality as baseline requirement) was incomplete without addressing multicollinearity. We have now fully addressed this issue.

 Specific changes made:

 •    Added Section 4.4.3 "Multicollinearity Assessment" before presenting H₃ results

 •    Added Table (16) showing Pearson correlations among IQ, SQ, and SEQ (r = 0.658-0.712)

 •    Added Table (17) showing VIF values for all predictors (1.96-2.14, all < 5.0, acceptable)

 •    Revised the discussion of system quality suppression to include both statistical (multicollinearity, shared variance) and theoretical (Herzberg's hygiene factor) explanations

 •    Added explicit statement that VIF values are within acceptable limits, but shared variance still affects coefficient estimates

 •    Added acknowledgment that the suppression of SQ is a statistical phenomenon (shared variance) that may also have theoretical meaning (hygiene factor)

 Comment #7 (Research Question 2 - Customer Awareness)

 Reviewer's Comment: "The second research question inquires about customer awareness of shopping store specifications, yet no hypothesis is developed to test this, and the results section ignores it entirely."

 Response: The reviewer is correct. Research question 2 was descriptive, not inferential, and therefore did not require a hypothesis. However, the results section did not adequately address it. We have now added explicit descriptive statistics to answer RQ2.

 Specific changes made:

 •    Added explicit statement in Section 1.5 that RQ2 is descriptive and will be answered using means and standard deviations

 •    Added to Section 4.3 (Descriptive Statistics) a specific comment addressing customer awareness levels

 •    Added interpretation that the mean score of 3.592/5 indicates "moderate awareness" with significant room for improvement

 •    Added a practical implication based on this finding

 •    Location in revised manuscript: Section 1.5 (page 7), Section 4.3 (page 24), Table (13)
